# Supplementary material for: Cellular arrangement impacts metabolic activity and antibiotic tolerance in Pseudomonas aeruginosa biofilms
Source: PLoS Biol. 2024 Feb 1;22(2):e3002205. doi: 10.1371/journal.pbio.3002205 (PMC10833521; doi:10.1371/journal.pbio.3002205)
Supplement: S1 Fig — PA14 WT constitutively expressing mScarlet (2.5%) was mixed with PA14 WT (97.5%), spotted on 1% tryptone 1% agar plates, and grown for 3 days. The percentages of fluorescent cells were determined after 3 days of growth by homogenizing the macrocolonies and plating for colony-forming units (CFUs). Approximately 2.5% of CFUs expressed mScarlet. Results for 4 biological replicates are shown. The data underlying this figure can be found in S1_raw_data. (PDF) [file pbio.3002205.s001.pdf]

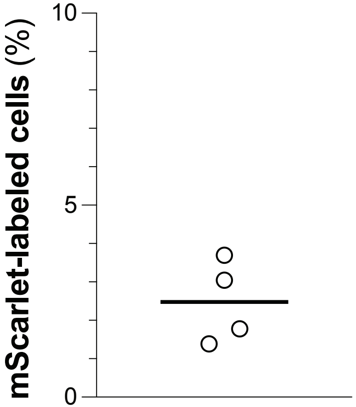

S1 Fig. Expression of mScarlet does not impact fitness in macrocolony biofilms. PA14 WT consitutively expressing mScarlet (2.5%) was mixed with PA14 WT (97.5%), spotted on 1% tryptone 1% agar plates, and grown for three days. The percentages of fluorescent cells were determined after three days of growth by homogenizing the macrocolonies and plating for colony-forming units (CFUs). ~2.5% of CFUs expressed mScarlet. Results for four biological replicates are shown. The data underlying this figure can be found in S1_raw_data.
