# Supplementary material for: Cellular arrangement impacts metabolic activity and antibiotic tolerance in Pseudomonas aeruginosa biofilms
Source: PLoS Biol. 2024 Feb 1;22(2):e3002205. doi: 10.1371/journal.pbio.3002205 (PMC10833521; doi:10.1371/journal.pbio.3002205)
Supplement: S2 Fig — Signal across depth detected for D2O in live two-day-old PA14 biofilms imaged by SRS. D2O is normalized to total protein. Results are shown for 3 biological replicates. The data underlying this figure can be found in S1_raw_data. (PDF) [file pbio.3002205.s002.pdf]

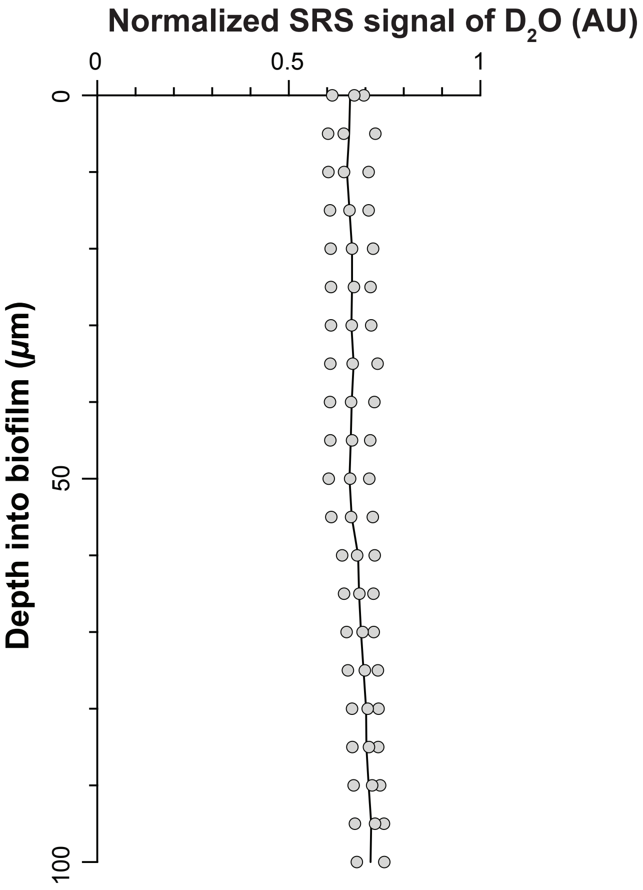

S2 Fig. D_2O distribution is uniform in macrocolony biofilms. Signal across depth detected for D_2O in live two-day-old PA14 biofilms imaged by SRS. D_2O is normalized to total protein. Results are shown for three biological replicates. The data underlying this figure can be found in S1_raw_data.
