# Supplementary material for: Cellular arrangement impacts metabolic activity and antibiotic tolerance in Pseudomonas aeruginosa biofilms
Source: PLoS Biol. 2024 Feb 1;22(2):e3002205. doi: 10.1371/journal.pbio.3002205 (PMC10833521; doi:10.1371/journal.pbio.3002205)
Supplement: S3 Fig — Fluorescence micrographs of thin sections from WT biofilms grown on 1% tryptone, 1% agar under oxic or anoxic conditions for 3 days. Where indicated, 40 mM potassium nitrate was included in the growth medium. Biofilm inocula contained 2.5% cells that constitutively express mScarlet. mScarlet fluorescence is colored yellow. Scale bar is 25 μm and applies to all images. (PDF) [file pbio.3002205.s003.pdf]

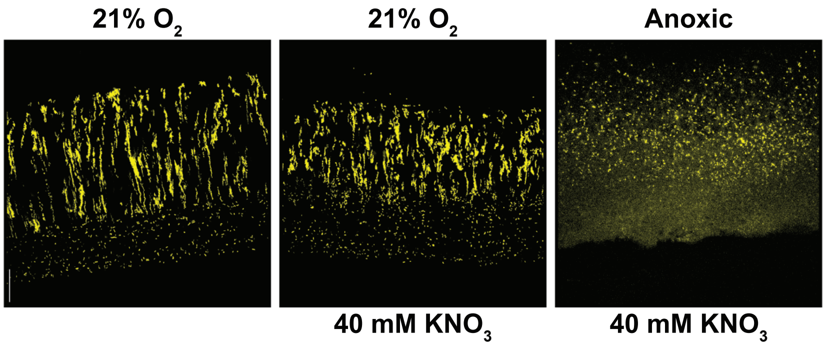


**S3 Fig. Anoxic conditions abrogate striation formation.** Fluorescence micrographs of thin sections from WT biofilms grown on 1% tryptone, 1% agar under oxic or anoxic conditions for 3 days. Where indicated, 40 mM potassium nitrate was included in the growth medium. Biofilm inocula contained 2.5% cells that constitutively express mScarlet. mScarlet fluorescence is colored yellow. Scale bar is 25 μm and applies to all images.
