# Supplementary material for: Cellular arrangement impacts metabolic activity and antibiotic tolerance in Pseudomonas aeruginosa biofilms
Source: PLoS Biol. 2024 Feb 1;22(2):e3002205. doi: 10.1371/journal.pbio.3002205 (PMC10833521; doi:10.1371/journal.pbio.3002205)
Supplement: S4 Fig — Biofilms were grown for 72 h on 1% tryptone, 1% agar medium containing the dyes Congo red and Coomassie blue. Scale bar applies to all images. (PDF) [file pbio.3002205.s004.pdf]

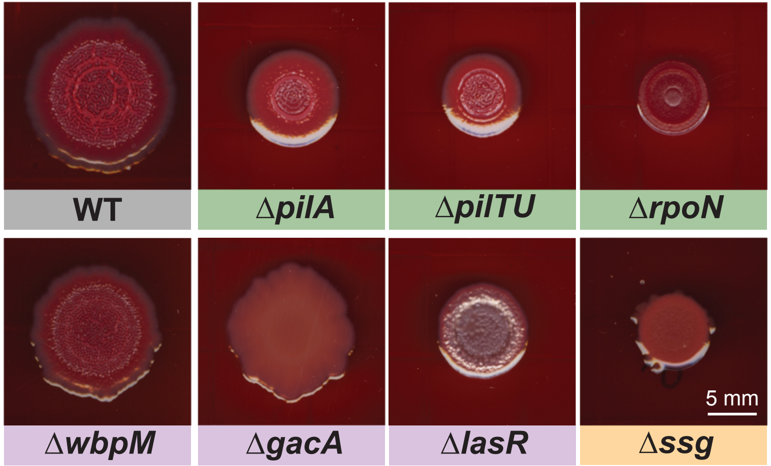

S4 Fig. Effects on cellular arrangement do not correlate with consistent effects on macrocolony morphology between mutants. Biofilms were grown for 72 h on 1% tryptone, 1% agar medium containing the dyes Congo red and Coomassie blue. Scale bar applies to all images.
