# Supplementary material for: Cellular arrangement impacts metabolic activity and antibiotic tolerance in Pseudomonas aeruginosa biofilms
Source: PLoS Biol. 2024 Feb 1;22(2):e3002205. doi: 10.1371/journal.pbio.3002205 (PMC10833521; doi:10.1371/journal.pbio.3002205)
Supplement: S5 Fig — Fluorescence micrographs of thin sections from indicated mutant biofilms grown on 1% tryptone and 1% agar for 3 days. The biofilm inocula contained constitutive mScarlet-expressers at a frequency of 2.5%; 97.5% of the cells did not express a fluorophore. Scale bar is 25 μm. Images are representative of at least 2 independent experiments and mScarlet fluorescence is colored yellow. Scale bar applies to all images. (PDF) [file pbio.3002205.s005.pdf]

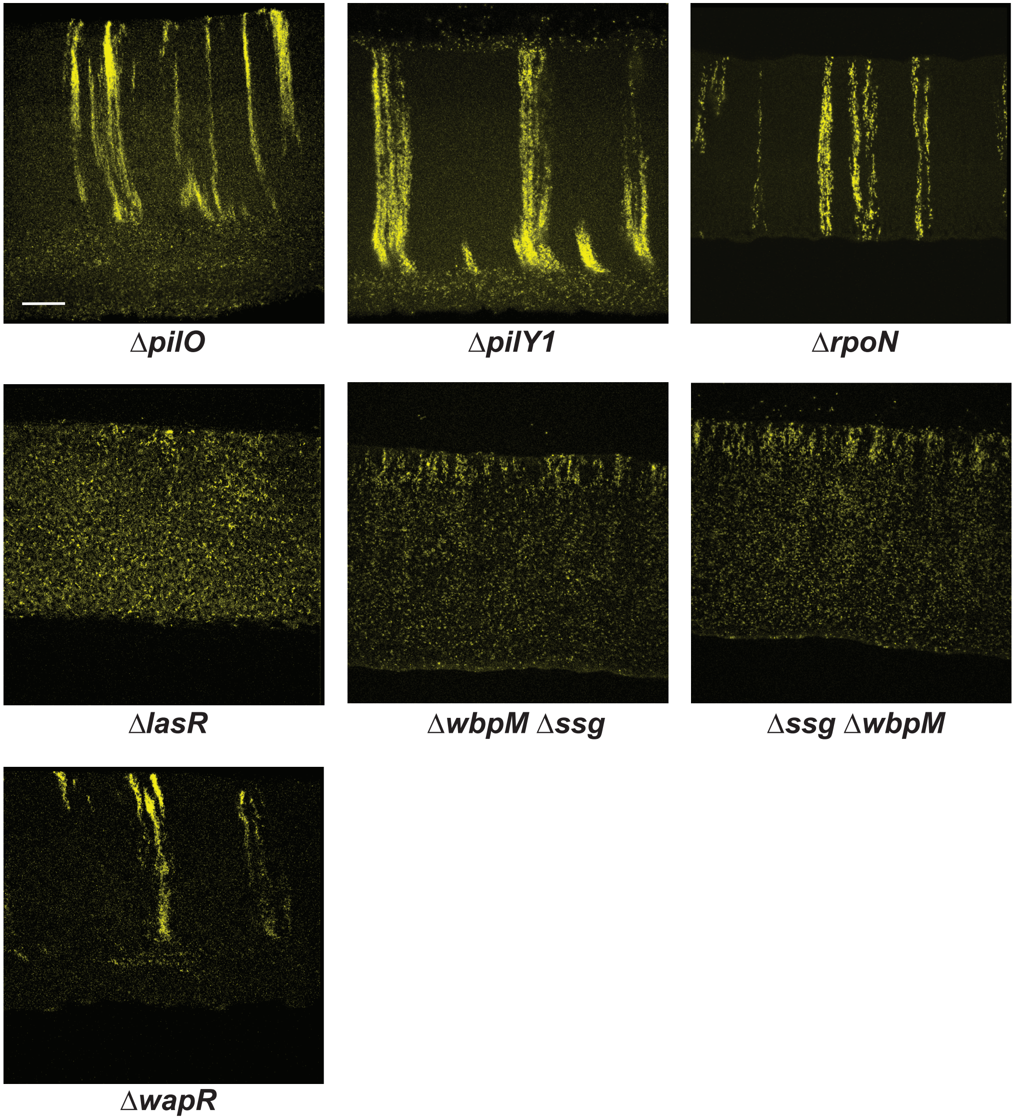

S5 Fig. Mutations affecting global regulators, pilus synthesis, and O-antigen synthesis/attachment alter cell patterning in colony biofilms. Fluorescence micrographs of thin sections from indicated mutant biofilms grown on 1% tryptone and 1% agar for three days. The biofilm inocula contained constitutive mScarlet-expressers at a frequency of 2.5%; 97.5% of the cells did not express a fluorophore. Scale bar is 25 µm. Images are representative of at least two independent experiments and mScarlet fluorescence is colored yellow. Scale bar applies to all images.
