# Supplementary material for: Cellular arrangement impacts metabolic activity and antibiotic tolerance in Pseudomonas aeruginosa biofilms
Source: PLoS Biol. 2024 Feb 1;22(2):e3002205. doi: 10.1371/journal.pbio.3002205 (PMC10833521; doi:10.1371/journal.pbio.3002205)
Supplement: S6 Fig — (A) Uncropped version of western blot shown in Fig 5B indicating PilA protein levels from sheared cells for macrocolony biofilms of WT and the indicated mutants. (B) Cropped western blot showing PilA protein levels from whole cells for macrocolony biofilms of WT and the indicated mutants. (C) Uncropped version of western blot shown in (B). (A–C) For each gel, equal amounts of total protein were loaded per lane and resolved by SDS-PAGE using a 15% polyacrylamide gel. The PilA protein was detected using an anti-PilA antibody. (PDF) [file pbio.3002205.s006.pdf]

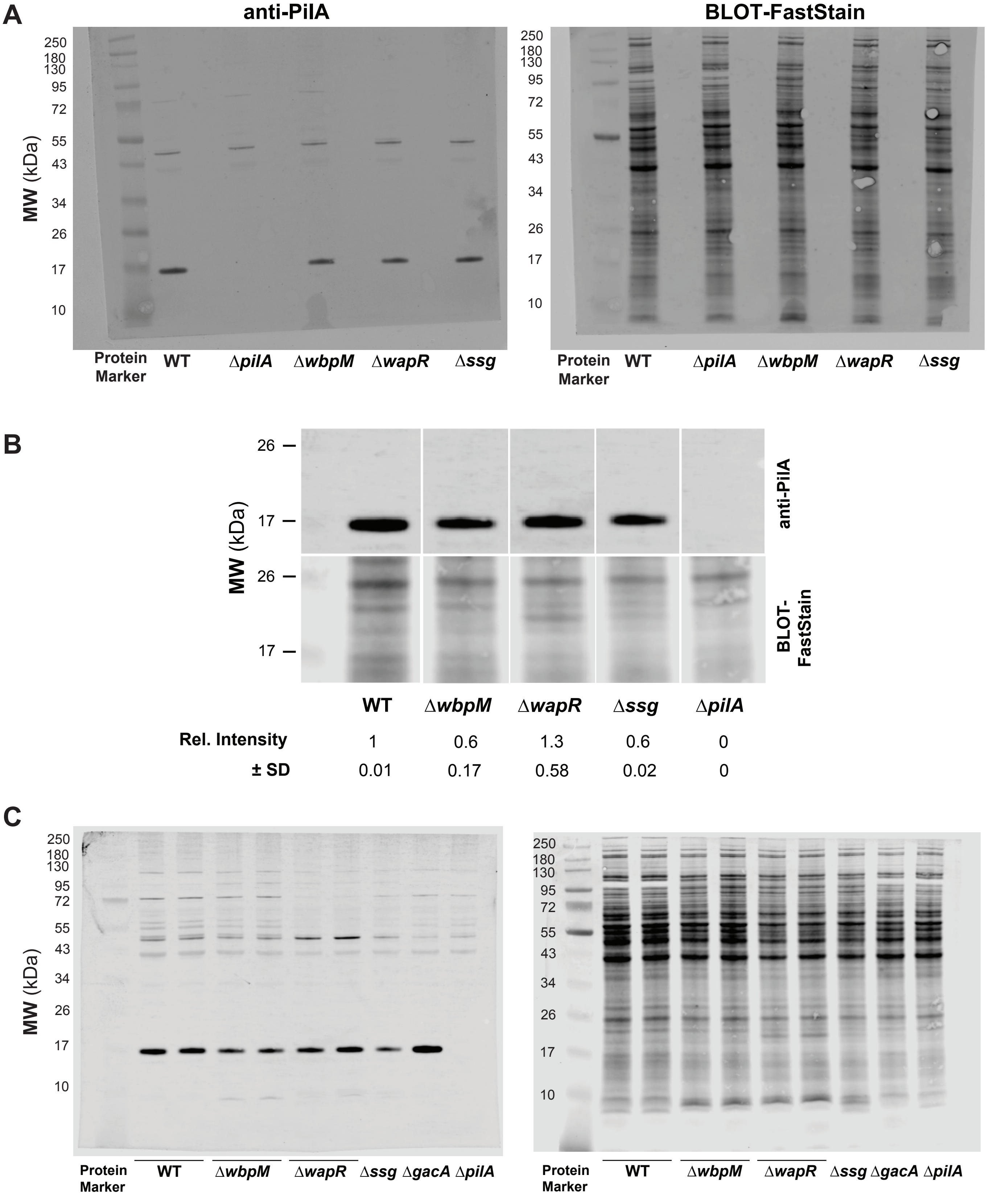

S6 Fig. Cell-arrangement phenotypes are not due to changes in PilA levels. (**A**) Uncropped version of Western blot shown in **Fig 5B** indicating PilA protein levels from sheared cells for macrocolony biofilms of WT and the indicated mutants. (**B**) Cropped Western blot showing PilA protein levels from whole cells for macrocolony biofilms of WT and the indicated mutants. (**C**) Uncropped version of Western blot shown in (B). (**A-C**) For each gel, equal amounts of total protein were loaded per lane and resolved by SDS-PAGE using a 15% polyacrylamide gel. The PilA protein was detected using an anti-PilA antibody.
