# Supplementary material for: Cellular arrangement impacts metabolic activity and antibiotic tolerance in Pseudomonas aeruginosa biofilms
Source: PLoS Biol. 2024 Feb 1;22(2):e3002205. doi: 10.1371/journal.pbio.3002205 (PMC10833521; doi:10.1371/journal.pbio.3002205)
Supplement: S7 Fig — Fluorescence micrographs of thin sections from WT and indicated mutant biofilms. Biofilm inocula contained 2.5% cells that constitutively express eGFP and 97.5% RhaSR-PrhaBAD-controlled mScarlet-producing strain. RhaSR-PrhaBAD-controlled mScarlet was induced with 0.5% L-rhamnose. Top panels show eGFP fluorescence (colored yellow) and bottom panels show the mScarlet fluorescence for each thin section. Quantification of mScarlet fluorescence is shown on the right. Scale bar is 25 μm and applies to all images. The data underlying this figure can be found in S1_raw_data. (PDF) [file pbio.3002205.s007.pdf]

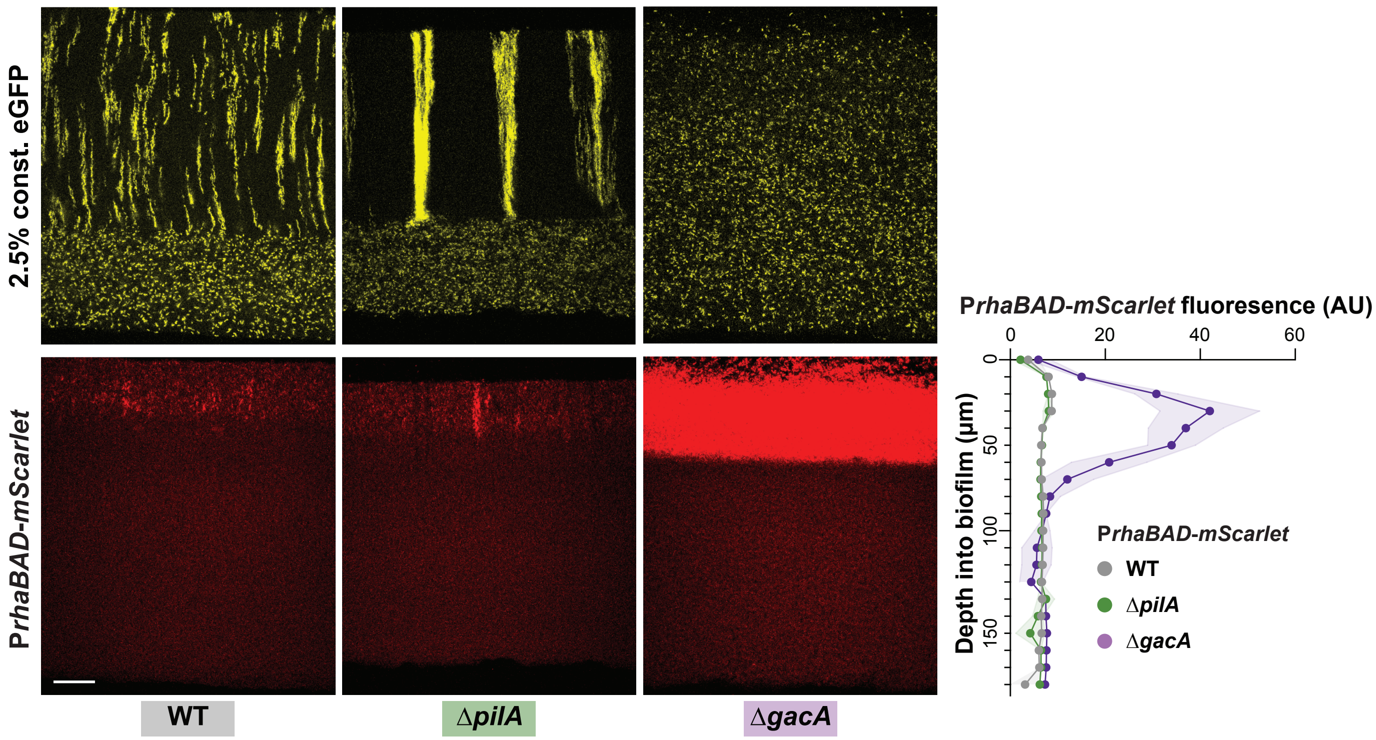

S7 Fig. Δ*gacA* biofilms show increased rhamnose-dependent expression at the biofilm-air interface, consistent with their disordered cellular arrangement. Fluorescence micrographs of thin sections from WT and indicated mutant biofilms. Biofilm inocula contained 2.5% cells that constitutively express eGFP and 97.5% RhaSR-*PrhaBAD*-controlled mScarlet-producing strain. RhaSR-*PrhaBAD*-controlled mScarlet was induced with 0.5% L-rhamnose. Top panels show eGFP fluorescence (colored yellow) and bottom panels show the mScarlet fluorescence for each thin section. Quantification of mScarlet fluorescence is shown on the right. Scale bar is 25 µm and applies to all images. The data underlying this figure can be found in S1_raw_data.
