# Supplementary material for: Cellular arrangement impacts metabolic activity and antibiotic tolerance in Pseudomonas aeruginosa biofilms
Source: PLoS Biol. 2024 Feb 1;22(2):e3002205. doi: 10.1371/journal.pbio.3002205 (PMC10833521; doi:10.1371/journal.pbio.3002205)
Supplement: S8 Fig — Changes in fluorescence (top) and optical density (bottom) for the indicated reporter strains during growth in liquid culture with or without the addition of 0.5% L-rhamnose. The data underlying this figure can be found in S1_raw_data. (PDF) [file pbio.3002205.s008.pdf]

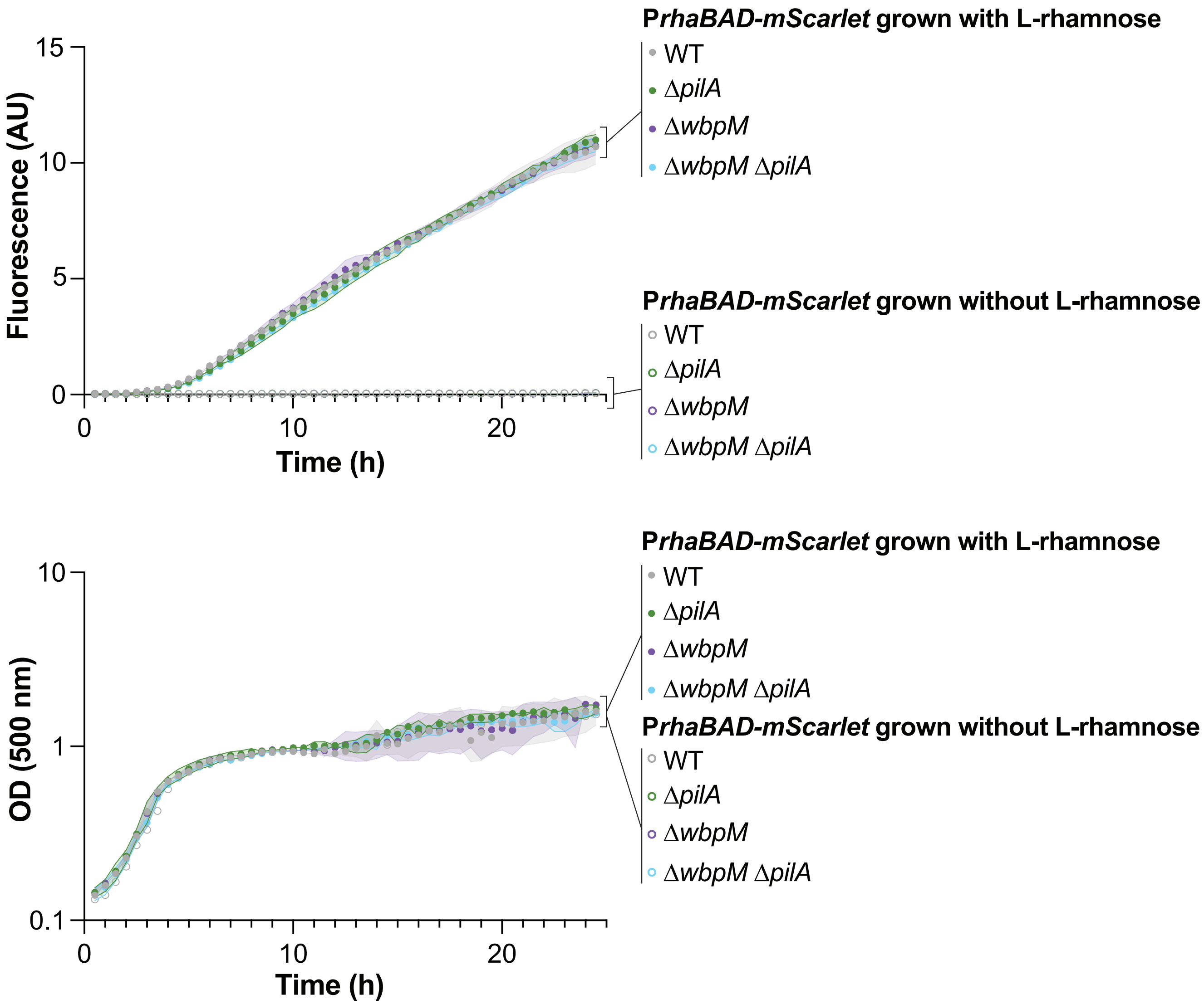

S8 Fig. RhaSR-*PrhaBAD*-controlled mScarlet production in WT, Δ*wbpM*, Δ*pilA*, Δ*wbpM* Δ*pilA* strains is comparable during planktonic growth. Changes in fluorescence (top) and optical density (bottom) for the indicated reporter strains during growth in liquid culture with or without the addition of 0.5% L-rhamnose. The data underlying this figure can be found in S1_raw_data.
