# Supplementary material for: Cellular arrangement impacts metabolic activity and antibiotic tolerance in Pseudomonas aeruginosa biofilms
Source: PLoS Biol. 2024 Feb 1;22(2):e3002205. doi: 10.1371/journal.pbio.3002205 (PMC10833521; doi:10.1371/journal.pbio.3002205)
Supplement: S9 Fig — (A) Side view of a WT P. aeruginosa pellicle biofilm grown for 3 days in a cuvette. WT and ΔwbpM form comparable pellicles. (B) Live light sheet microscopy images of WT and ΔwbpM mixing assay pellicle biofilms. Scale bar applies to both images. (C) Quantification of Sulfo-Cy5 NHS-ester fluorescence after addition to the medium below each pellicle biofilm. The signal was normalized by total sum. The data underlying this figure can be found in S1_raw_data. (PDF) [file pbio.3002205.s009.pdf]

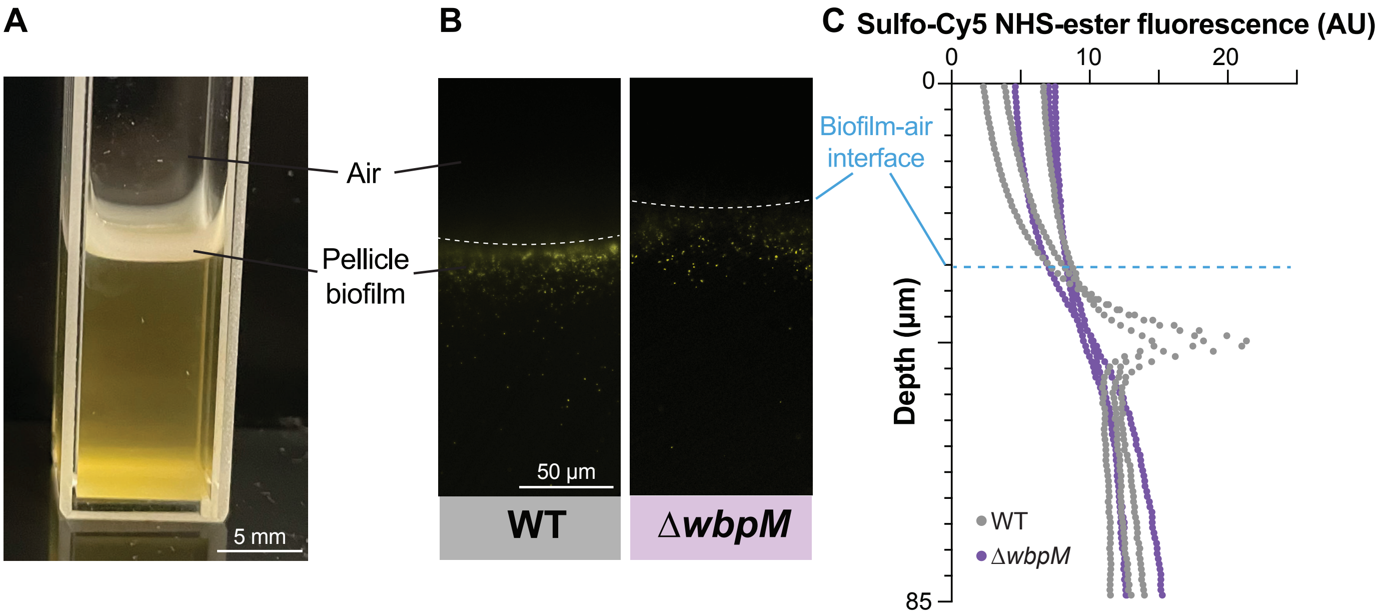

S9 Fig. Dye distribution in live pellicle biofilm. (**A**) Side view of a WT *P. aeruginosa* pellicle biofilm grown for three days in a cuvette. WT and Δ*wbpM* form comparable pellicles. (**B**) Live light-sheet microscopy images of WT and Δ*wbpM* mixing assay pellicle biofilms. Scale bar applies to both images. (**C**) Quantification of Sulfo-Cy5 NHS-ester fluorescence after addition to the medium below each pellicle biofilm. The signal was normalized by total sum. The data underlying this figure can be found in S1_raw_data.
