# Supplementary material for: Cellular arrangement impacts metabolic activity and antibiotic tolerance in Pseudomonas aeruginosa biofilms
Source: PLoS Biol. 2024 Feb 1;22(2):e3002205. doi: 10.1371/journal.pbio.3002205 (PMC10833521; doi:10.1371/journal.pbio.3002205)
Supplement: S10 Fig — SRS micrographs of WT and Δssg biofilm thin sections. Scale bar is 25 μm and applies to both images. The data underlying this figure can be found in S1_raw_data. (PDF) [file pbio.3002205.s010.pdf]

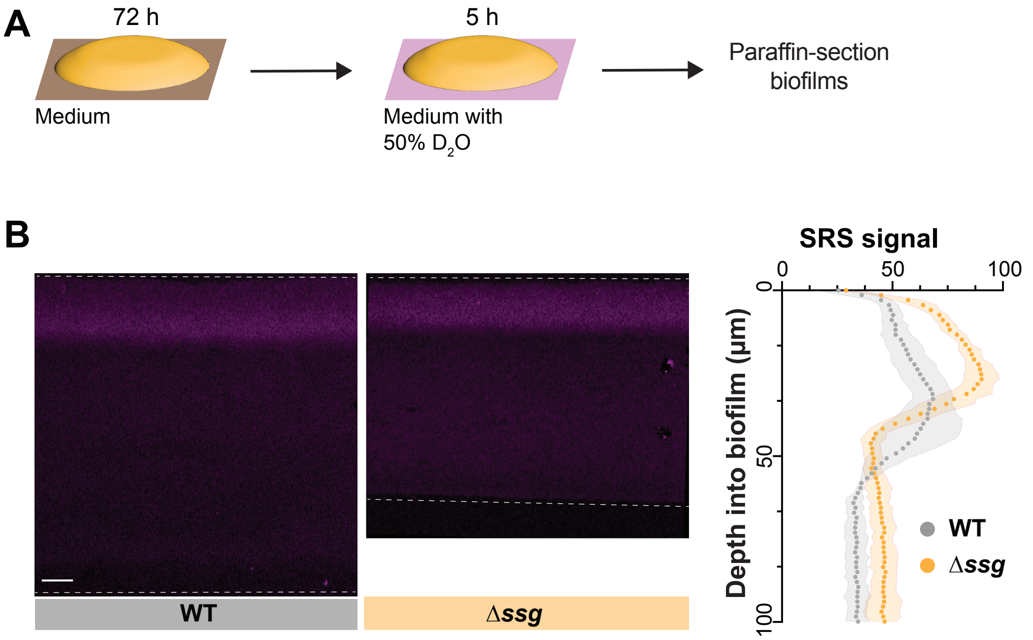

S10 Fig. Metabolic activity profile within Δ*ssg* biofilm. (**A**) Schematic illustration of the experimental setup for growing *P. aeruginosa* biofilms on agar plates and their subsequent transfer to a medium containing 50% D_2O. (**B**) SRS micrographs of WT and Δ*ssg* biofilm thin-sections. Scale bar is 25 μm and applies to both images. The data underlying this figure can be found in S1_raw_data.
