# Supplementary material for: Cellular arrangement impacts metabolic activity and antibiotic tolerance in Pseudomonas aeruginosa biofilms
Source: PLoS Biol. 2024 Feb 1;22(2):e3002205. doi: 10.1371/journal.pbio.3002205 (PMC10833521; doi:10.1371/journal.pbio.3002205)
Supplement: S11 Fig — (A) Fluorescence micrographs of thin sections from indicated mutant biofilms grown on 1% tryptone and 1% agar for 3 days. The biofilm inocula contained 2.5% of cells that constitutively express mScarlet (shown as yellow); 97.5% did not express a fluorophore. Scale bar is 25 μm and applies to all images. (B) Schematic illustration of the experimental setup for growing P. aeruginosa biofilms on agar plates and their subsequent transfer to a medium containing 50 μm propidium iodide or 50 μm propidium iodide + 100 μg/ml tobramycin. (C) Left: Fluorescence micrographs of 3-day-old biofilms exposed to propidium iodide. Center: Fluorescence micrographs of biofilms treated with tobramycin and PI. Right: Graphs showing quantification of PI staining. Images shown in this figure are representative of at least 2 independent experiments. The data underlying this figure can be found in S1_raw_data. (PDF) [file pbio.3002205.s011.pdf]

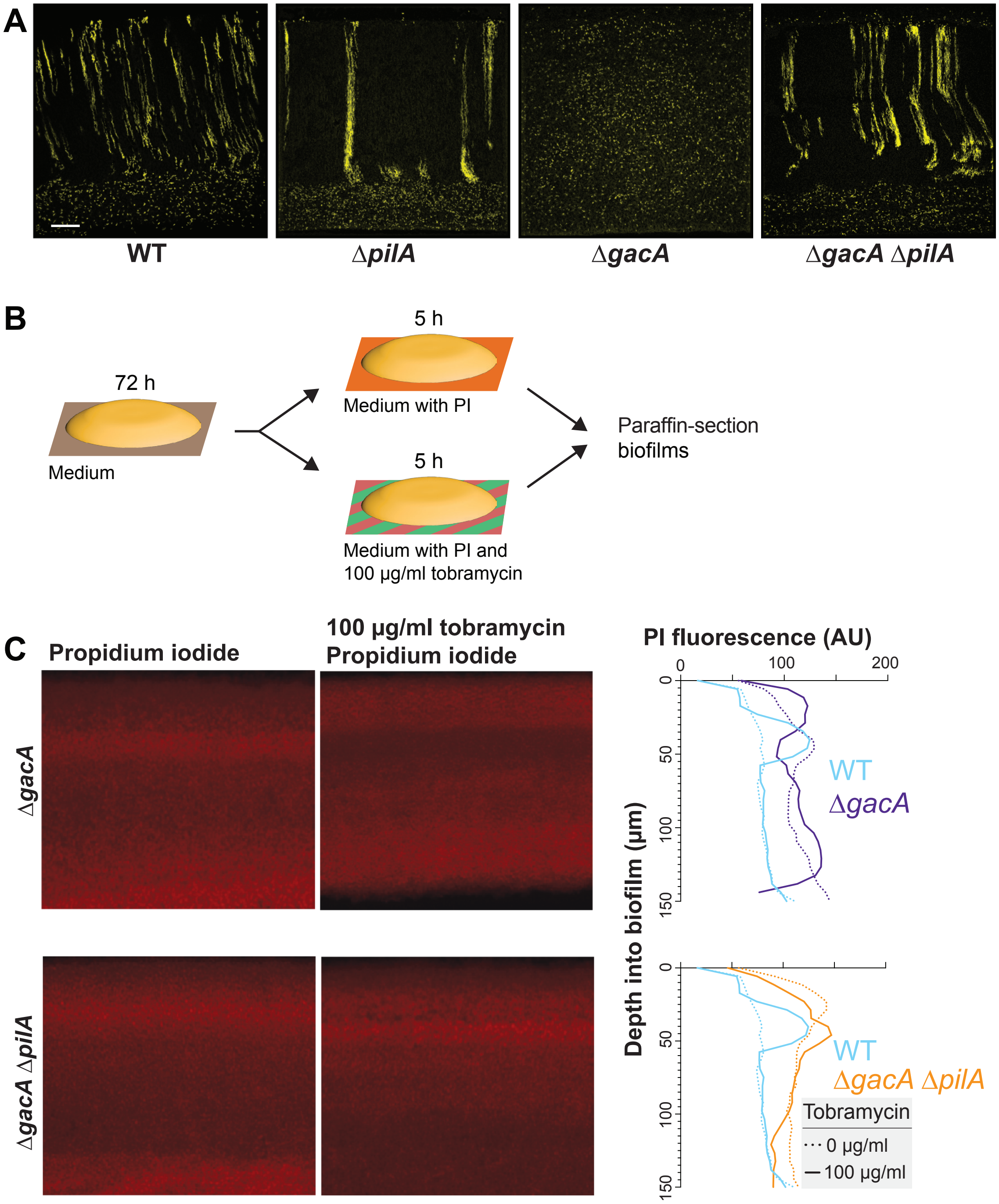

S11 Fig. Mutations affecting cellular arrangement alter biofilm antibiotic tolerance profiles. (**A**) Fluorescence micrographs of thin sections from indicated mutant biofilms grown on 1% tryptone and 1% agar for three days. The biofilm inocula contained 2.5% of cells that constitutively express mScarlet (shown as yellow); 97.5% did not express a fluorophore. Scale bar is 25 µm and applies to all images. (**B**) Schematic illustration of the experimental setup for growing *P. aeruginosa* biofilms on agar plates and their subsequent transfer to a medium containing 50 µM propidium iodide or 50 µM propidium iodide + 100 µg/mL tobramycin. (**C**) **Left**: Fluorescence micrographs of 3-day-old biofilms exposed to propidium iodide. **Center**: Fluorescence micrographs of biofilms treated with tobramycin and PI. **Right**: Graphs showing quantification of PI staining. Images shown in this figure are representative of at least two independent experiments. The data underlying this figure can be found in S1_raw_data.
