# Supplementary material for: Cellular arrangement impacts metabolic activity and antibiotic tolerance in Pseudomonas aeruginosa biofilms
Source: PLoS Biol. 2024 Feb 1;22(2):e3002205. doi: 10.1371/journal.pbio.3002205 (PMC10833521; doi:10.1371/journal.pbio.3002205)
Supplement: S1 Table — (PDF) [file pbio.3002205.s012.pdf]

Table S1. Bacterial strains used in this study.

Number	Strain	Description	Source
Pseudomonas aeruginosa strains			
LD0	UCBPP-PA14 (WT)	Clinical isolate UCBPP-PA14.	[1]
LD4058	PA14 Δ*vfr*	PA14 with *vfr (PA14_08370)* deleted. Made by mating pLD4042 into LD0.	This study
LD732	PA14 Δ*pqsA-C*	PA14 with *pqsA-C (PA14_51430*, *PA14_51420*, and *PA14_51410)* deleted.	[2]
LD1380	PA14 Δ*rhlR*	PA14 with *rhlR (PA14_19120)* deleted. Made by mating pLD1355 into LD0.	[2]
LD4555	PA14 Δ*antABC*	PA14 with *antABC (PA14_32160*, *PA14_32150*, and *PA14_32140) deleted*. Made by mating pLD4537 into LD0.	This study
LD24	PA14 Δ*phz* (a.k.a. Δ*phz1/2*)	PA14 with *phz (PA14_09480-PA14_09410* and *PA14_39970-PA14_39880)* operons deleted.	[2]
LD3679	PA14 Δ*gacA*	PA14 with *gacA (PA14_30650)* deleted. Made by mating pLD3079 into LD0.	This study
LD4213	PA14 Δ*lasR*	PA14 with *lasR (PA14_45960)* deleted. Made by mating pLD4228 into LD0.	This study
LD3596	PA14 Δ*aer1*	PA14 with *aer1 (PA14_44300)* deleted. Made by mating pLD3594 into LD0.	This study
LD3604	PA14 Δ*aer2*	PA14 with *aer2 (PA14_02220)* deleted. Made by mating pLD3579 into LD0.	This study
LD3607	PA14 Δ*aer1* Δ*aer2*	PA14 with *aer1* and *aer2 (PA14_44300* and *PA14_02220)* deleted. Made by mating pLD3594 into LD3604.	This study
LD2271	PA14 Δ*nosP*	PA14 with *nosP (PA14_38990 and PA14_38970)* deleted. Made by mating LD2271 into LD0.	This study
LD915	PA14 Δ*anr*	PA14 with *anr* (*PA14_44490)* deleted.	[3]
LD369	PA14 Δ*pilB*	PA14 with *pilB (PA14_58750)* deleted.	[4]
LD1879	PA14 Δ*pilY1*	PA14 with *pilY1 (PA14_60310)* deleted. Made by mating pLD1858 into LD0.	This study
LD3986	PA14 Δ*pilA*	PA14 with *pilA (PA14_58730)* deleted. Made by mating pLD3979 into LD0.	This study
LD4164	PA14 Δ*pilT* Δ*pilU*	PA14 with *pilT pilU (PA14_05180 and PA14_05190)* deleted. Made by mating pLD4137 into LD0.	This study
LD4644	PA14 Δ*ssg*	PA14 with *ssg (PA14_66120)* deleted. Made by mating pLD4630 into LD0.	This study
LD4913	PA14 Δ*wapR*	PA14 with *wapR (PA14_66110)* deleted. Made by mating pLD4832 into LD0.	This study
LD4635	PA14 Δ*wbpM*	PA14 with *wbpM (PA14_23470)* deleted. Made by mating pLD4631 into LD0.	This study
LD3950	PA14 Δ*cheY*	PA14 with *cheY (PA14_45620)* deleted. Made by mating pLD3939 into LD0.	This study
LD371	PA14 Δ*flgK*	PA14 with *flgK (PA14_50360)* deleted.	[4]
LD384	PA14 Δ*pilb* Δ*flgK*	PA14 with *pilb* and *flgK (PA14_58750* and *PA14_503*60) deleted.	[4]
LD3621	PA14 Δ*motA* Δ*motB*	PA14 with *motA* and *motB* (*PA14_65450* and *PA14_65430)* deleted. Made by mating pLD3629 into LD0.	This study

LD3622	PA14 ∆*motC* ∆*motD*	PA14 with *motC and motD (PA14_45560* and *PA14_45540)* deleted. Made by mating pLD3630 into LD0.	This study
LD1529	PA14 ∆*cupA2*	PA14 with *cupA2 (PA14_37040)* deleted. Made by mating pLD1547 into LD0.	This study
LD1726	PA14 ∆cupA ∆*cupD*	PA14 with *cupD1 (PA14_59710)* deleted. Made by mating pLD1704 into LD1529.	This study
LD3070	PA14 ∆*pelA-G*	PA14 with *pelA-G (PA14_24480*, *PA14_24490*, *PA14_24500*, *PA14_24510*, *PA14_24530*, *PA14_24550*, and *PA14_24560)* deleted. Made by mating pLDLD3059 into LD0.	This study
LD3192	PA14 ∆*rpoS*	PA14 with *rpoS (PA14_17480)* deleted. Made by mating pLD3471 into LD0.	[5]
LD3190	PA14 ∆*rpoN*	PA14 with *rpoN (PA14_57940)* deleted. Made by mating pLD3473 into LD0.	[5]
LD3949	PA14 ∆*fliA*	PA14 with *fliA (PA14_45630)* deleted. Made by mating pLD3938 into LD0.	This study
LD3674	PA14 ∆*crc*	PA14 with *crc (PA14_70390)* deleted.	[5]
LD3130	PA14 ∆*ptsP*	PA14 with *ptsP (PA14_04410)* deleted. Made by mating pLD3125 into LD0.	This study
LD3694	PA14 ∆*ptsO*	PA14 with *ptsO* (*PA14_57980)* deleted. Made by mating pLD3635 into LD0.	This study
LD3696	PA14 ∆*ptsO* ∆*ptsP*	PA14 with *ptsO* and *ptsP (PA14_04410* and *PA14_57980)* deleted. Made by mating pLD3635 into LD3130.	This study
LD1888	PA14 ∆*ccoN1* ∆*ccoN2*	PA14 with *ccoN1* and *ccoN2 (PA14_44370* and *PA14_44340)* deleted. Made by mating pLD1610 into LD1784.	[6]
LD1976	PA14 ∆*ccoN1* ∆*ccoN2* ∆*ccoN4*	PA14 with *ccoN1*, *ccoN2*, and *ccoN4 (PA14_443470*, *PA14_44340*, and *PA14_10500)* deleted. Made by mating pLD1264 into LD1888.	[6]
LD1933	PA14 ∆*cco1* ∆*cco2*	PA14 with both *cco* operons *(PA14_44340-PA14_44400*) deleted simultaneously.	[6]
LD3196	PA14 ∆*dipA*	PA14 with *dipA (PA14_66320)* deleted. Made by mating pLD1204 into LD0.	This study
LD4687	PA14 ∆*relA*	PA14 with *relA (PA14_52180)* deleted. Made by mating pLD4642 into LD0.	[7]
LD2177	PA14 ∆*sadC*	PA14 with *sadC (PA14_56280)* deleted. Made by mating pLD2173 into LD0.	[7]
LD2569	PA14 ∆*bifA*	PA14 with *bifA (PA14_56790)* deleted. Made by mating pLD2565 into LD0.	[7]
LD2183	PA14 ∆*roeA*	PA14 with *roeA (PA14_50060)* deleted. Made by mating pLD2179 into LD0.	[7]
LD2227	PA14 ∆*rmcA*	PA14 with *rmcA* (*PA14_07500)* deleted. Made by mating pLD909 into LD0.	[7]
LD2428	PA14 ∆*wspR*	PA14 with *wspR* (*PA14_16500)* deleted. Made by mating pLD4040 into LD0.	[7]
LD3917	PA14 ∆*cyaA*	PA14 with *cyaA (PA14_69610)* deleted. Made by mating pLD3910 into LD0.	This study
LD3920	PA14 ∆*cyaB*	PA14 with *cyaB (PA14_22620)* deleted. Made by mating pLD3911 into LD0.	This study
LD3923	PA14 ∆*cyaA* ∆*cyaB*	PA14 with *cyaA cyaB (PA14_69610* and *PA14_22620)* deleted. Made by mating pLD3910 into LD3920 LD0.	This study
LD3914	PA14 ∆*cpdA*	PA14 with *cpdA (PA14_65690)* deleted. Made by mating pLD3909 into LD0.	This study
LD3625	PA14 ∆*ackA*	PA14 with *ackA (PA14_53470)* deleted. Made by mating pLD3609 into LD0.	This study

LD2729	PA14 Δ*ldhA*	PA14 with *ldhA (PA14_52270)* deleted. Made by mating pLD2728 into LD0.	[8]
LD3646	PA14 Δ*ldhA* Δ*ackA*	PA14 with *ldhA ackA (PA14_53470* and *PA14_52270)* deleted. Made by mating pLD2728 into LD3625.	This study
LD4833	PA14 Δ*pilA* Δ*ssg*	PA14 with *pilA ssg (PA14_58730* and *PA14_66120)* deleted. Made by mating pLD4630 into LD3986.	This study
LD4836	PA14 Δ*pilA* Δ*wbpM*	PA14 with *pilA wbpM (PA14_58730* and *PA14_23470)* deleted. Made by mating pLD4631 into LD3986.	This study
LD4839	PA14 Δ*ssg* Δ*wbpM*	PA14 with *ssg wbpM (PA14_66120* and *PA14_23470)* deleted. Made by mating pLD4631 into LD4644.	This study
LD4835	PA14 Δ*wbpM* Δ*ssg*	PA14 with *wbpM ssg (PA14_23470* and *PA14_66120)* deleted. Made by mating pLD4630 into LD4635.	This study
LD4764	PA14 $P_{A1/04/03}$-mScarlet	PA14 constitutively expressing mScarlet. Made by mating pLD3433 into LD0.	This study
LD4070	PA14 Δ*vfr* $P_{A1/04/03}$-mScarlet	PA14 Δ*vfr* constitutively expressing mScarlet. Made by mating pLD3433 into LD4058.	This study
LD4007	PA14 Δ*pqsA-C* $P_{A1/04/03}$-mScarlet	PA14 Δ*pqsA-C* constitutively expressing mScarlet. Made by mating pLD3433 into LD732.	This study
LD4008	PA14 Δ*rhlR* $P_{A1/04/03}$-mScarlet	PA14 Δ*rhlR* constitutively expressing mScarlet. Made by mating pLD3433 into LD1380.	This study
LD4765	PA14 Δ*antABC* $P_{A1/04/03}$-mScarlet	PA14 Δ*antABC* constitutively expressing mScarlet. Made by mating pLD3433 into LD4555.	This study
LD3608	PA14 Δ*phz* (Δ*phz1/2*) $P_{A1/04/03}$-mScarlet	PA14Δ*phz* constitutively expressing mScarlet. Made by mating pLD3433 into LD24.	This study
LD5074	PA14 Δ*gacA* $P_{A1/04/03}$-mScarlet	PA14 Δ*gacA* constitutively expressing mScarlet. Made by mating pLD3433 into LD3679.	This study
LD4763	PA14 Δ*lasR* $P_{A1/04/03}$-mScarlet	PA14 Δ*lasR* constitutively expressing mScarlet. Made by mating pLD3433 into LD4213.	This study
LD5075	PA14 Δ*aer1* $P_{A1/04/03}$-mScarlet	PA14 Δ*aer1* constitutively expressing mScarlet. Made by mating pLD3433 into LD3596.	This study
LD5076	PA14 Δ*aer2* $P_{A1/04/03}$-mScarlet	PA14 Δ*aer2* constitutively expressing mScarlet. Made by mating pLD3433 into LD3604.	This study
LD5077	PA14 Δ*aer1* Δ*aer2* $P_{A1/04/03}$-mScarlet	PA14 Δ*aer1* Δ*aer2* constitutively expressing mScarlet. Made by mating pLD3433 into LD3607.	This study
LD4217	PA14 Δ*nosP* $P_{A1/04/03}$-mScarlet	PA14 Δ*nosP* constitutively expressing mScarlet. Made by mating pLD3433 into LD.	This study
LD4127	PA14 Δ*anr* $P_{A1/04/03}$-mScarlet	PA14 Δ*anr* constitutively expressing mScarlet. Made by mating pLD3433 into LD915.	This study
LD5078	PA14 Δ*pilB* $P_{A1/04/03}$-mScarlet	PA14 Δ*pilB* constitutively expressing mScarlet. Made by mating pLD3433 into LD369.	This study
LD5079	PA14 Δ*pilY1* $P_{A1/04/03}$-mScarlet	PA14 Δ*pilY1* constitutively expressing mScarlet. Made by mating pLD3433 into LD1879.	This study

LD3987	PA14 Δ*pilA* $P_{A1/04/03}$-mScarlet	PA14 Δ*pilA* constitutively expressing mScarlet. Made by mating pLD3433 into LD3986.	This study
LD4175	PA14 Δ*pilT* Δ*pilU* $P_{A1/04/03}$-mScarlet	PA14 Δ*pilT* Δ*pilU* constitutively expressing mScarlet. Made by mating pLD3433 into LD4164.	This study
LD4665	PA14 Δ*ssg* $P_{A1/04/03}$-mScarlet	PA14 Δ*ssg* constitutively expressing mScarlet. Made by mating pLD3433 into LD4644.	This study
LD4921	PA14 Δ*wapR* $P_{A1/04/03}$-mScarlet	PA14 Δ*wapR* constitutively expressing mScarlet. Made by mating pLD3433 into LD4913.	This study
LD4666	PA14 Δ*wbpM* $P_{A1/04/03}$-mScarlet	PA14 Δ*wbpM* constitutively expressing mScarlet. Made by mating pLD3433 into LD4635.	This study
LD3953	PA14 Δ*cheY* $P_{A1/04/03}$-mScarlet	PA14 Δ*cheY* constitutively expressing mScarlet. Made by mating pLD3433 into LD3950.	This study
LD5080	PA14 Δ*flgK* $P_{A1/04/03}$-mScarlet	PA14 Δ*flgK* constitutively expressing mScarlet. Made by mating pLD3433 into LD371.	This study
LD5081	PA14 Δ*pilb* Δ*flgK* $P_{A1/04/03}$-mScarlet	PA14 Δ*pilb* Δ*flgK* constitutively expressing mScarlet. Made by mating pLD3433 into LD384.	This study
LD5082	PA14 Δ*motA* Δ*motB* $P_{A1/04/03}$-mScarlet	PA14 Δ*motA* Δ*motB* constitutively expressing mScarlet. Made by mating pLD3433 into LD3621.	This study
LD5083	PA14 Δ*motC* Δ*motD* $P_{A1/04/03}$-mScarlet	PA14 Δ*motC* Δ*motD* constitutively expressing mScarlet. Made by mating pLD3433 into LD3622.	This study
LD5084	PA14 Δ*cupA2* $P_{A1/04/03}$-mScarlet	PA14 Δ*cupA2* constitutively expressing mScarlet. Made by mating pLD3433 into LD1529.	This study
LD5085	PA14 Δ*cupA* Δ*cupD* $P_{A1/04/03}$-mScarlet	PA14 Δ*cupA* Δ*cupD* constitutively expressing mScarlet. Made by mating pLD3433 into LD1726.	This study
LD5086	PA14 Δ*pelA-G* $P_{A1/04/03}$-mScarlet	PA14 Δ*pelA-G* constitutively expressing mScarlet. Made by mating pLD3433 into LD3070.	This study
LD5087	PA14 Δ*rpoS* $P_{A1/04/03}$-mScarlet	PA14 Δ*rpoS* constitutively expressing mScarlet. Made by mating pLD3433 into LD3192.	This study
LD5088	PA14 Δ*rpoN* $P_{A1/04/03}$-mScarlet	PA14 Δ*rpoN* constitutively expressing mScarlet. Made by mating pLD3433 into LD3190.	This study
LD3951	PA14 Δ*fliA* $P_{A1/04/03}$-mScarlet	PA14 Δ*fliA* constitutively expressing mScarlet. Made by mating pLD3433 into LD3949.	This study
LD5089	PA14 Δ*crc* $P_{A1/04/03}$-mScarlet	PA14 Δ*crc* constitutively expressing mScarlet. Made by mating pLD3433 into LD3674.	This study
LD5090	PA14 Δ*ptsP* $P_{A1/04/03}$-mScarlet	PA14 Δ*ptsP* constitutively expressing mScarlet. Made by mating pLD3433 into LD3130.	This study
LD4008	PA14 Δ*ptsO* $P_{A1/04/03}$-mScarlet	PA14 Δ*ptsO* constitutively expressing mScarlet. Made by mating pLD3433 into LD3694.	This study

LD4009	PA14 Δ*ptsO* Δ*ptsP* $P_{A1/04/03}$-mScarlet	PA14 Δ*ptsO* Δ*ptsP* constitutively expressing mScarlet. Made by mating pLD3433 into LD3696.	This study
LD2013	PA14 Δ*ccoN1* Δ*ccoN2* $P_{A1/04/03}$-YFP	PA14 Δ*ccoN1* Δ*ccoN2* constitutively expressing YFP. Made by mating pLD68 into LD1888.	[6]
LD2136	PA14 Δ*ccoN1* Δ*ccoN2* Δ*ccoN4* $P_{A1/04/03}$-YFP	PA14 Δ*ccoN1* Δ*ccoN2* Δ*ccoN4* constitutively expressing YFP. Made by mating pLD68 into LD1976.	[6]
LD2012	PA14 Δ*cco1* Δ*cco2* $P_{A1/04/03}$-YFP	PA14 Δ*cco1* Δ*cco* constitutively expressing YFP. Made by mating pLD68 into LD1933.	[6]
LD5091	PA14 Δ*dipA* $P_{A1/04/03}$-mScarlet	PA14 Δ*dipA* constitutively expressing mScarlet. Made by mating pLD3433 into LD3196.	This study
LD4766	PA14 Δ*relA* $P_{A1/04/03}$-mScarlet	PA14 Δ*relA* constitutively expressing mScarlet. Made by mating pLD3433 into LD4687.	This study
LD2925	PA14 Δ*sadC* $P_{A1/04/03}$-YFP	PA14 Δ*sadC* constitutively expressing YFP. Made by mating pLD68 into LD2177.	This study
LD2773	PA14 Δ*bifA* $P_{A1/04/03}$-YFP	PA14 Δ*bifA* constitutively expressing YFP. Made by mating pLD68 into LD2569.	This study
LD2183	PA14 Δ*roeA* $P_{A1/04/03}$-YFP	PA14 Δ*roeA* constitutively expressing YFP. Made by mating pLD68 into LD2183.	This study
LD2775	PA14 Δ*rmcA* $P_{A1/04/03}$-YFP	PA14 Δ*rmcA* constitutively expressing YFP. Made by mating pLD68 into LD2227.	This study
LD2488	PA14 Δ*wspR* $P_{A1/04/03}$-YFP	PA14 Δ*wspR* constitutively expressing YFP. Made by mating pLD68 into LD2428.	This study
LD4069	PA14 Δ*cyaA* Δ*cyaB* $P_{A1/04/03}$-mScarlet	PA14 Δ*cyaA* Δ*cyaB* constitutively expressing mScarlet. Made by mating pLD3433 into LD3923.	This study
LD4062	PA14 Δ*cpdA* $P_{A1/04/03}$-mScarlet	PA14 Δ*cpdA* constitutively expressing mScarlet. Made by mating pLD3433 into LD3914.	This study
LD5092	PA14 Δ*ackA* $P_{A1/04/03}$-mScarlet	PA14 Δ*ackA* constitutively expressing mScarlet. Made by mating pLD3433 into LD3625.	This study
LD5093	PA14 Δ*ldhA* $P_{A1/04/03}$-mScarlet	PA14 Δ*ldhA* constitutively expressing mScarlet. Made by mating pLD3433 into LD2729.	This study
LD5094	PA14 Δ*pilA* Δ*ssg* $P_{A1/04/03}$-mScarlet	PA14 Δ*pilA* Δ*ssg* constitutively expressing mScarlet. Made by mating pLD3433 into LD3646.	This study
LD4850	PA14 Δ*pilA* Δ*wbpM* $P_{A1/04/03}$-mScarlet	PA14 Δ*pilA* Δ*wbpM* constitutively expressing mScarlet. Made by mating pLD3433 into LD4833.	This study
LD4853	PA14 Δ*ldhA* Δ*ackA* $P_{A1/04/03}$-mScarlet	PA14 Δ*ldhA* Δ*ackA* constitutively expressing mScarlet. Made by mating pLD3433 into LD4836.	This study
LD4852	PA14 Δ*wbpM* Δ*ssg* $P_{A1/04/03}$-mScarlet	PA14 Δ*wbpM* Δ*ssg* constitutively expressing mScarlet. Made by mating pLD3433 into LD4835.	This study

LD4855	PA14 Δ*ssg* Δ*wbpM* $P_{A1/04/03}$-mScarlet	PA14 Δ*ssg* Δ*wbpM* constitutively expressing mScarlet. Made by mating pLD3433 into LD4839.	This study
LD3801	PA14 WT $P_{A1/04/03}$-eGFP	PA14 constitutively expressing eGFP. Made by mating pLD3655 into LD0.	This study
LD4292	PA14 Δ*pilA* $P_{A1/04/03}$-eGFP	PA14 constitutively expressing eGFP. Made by mating pLD3655 into LD3986.	This study
LD4842	PA14 Δ*wbpM* $P_{A1/04/03}$-eGFP	PA14 constitutively expressing eGFP. Made by mating pLD3655 into LD4635.	This study
LD5047	PA14 Δ*wbpM* Δ*pilA* $P_{A1/04/03}$-eGFP	PA14 constitutively expressing eGFP. Made by mating pLD3655 into LD4836.	This study
LD4291	PA14 Δ*gacA* $P_{A1/04/03}$-eGFP	PA14 constitutively expressing eGFP. Made by mating pLD3655 into LD3679.	This study
LD4373	PA14 WT attB::rhaSR-PrhaBAD-mScarlet	PA14 attB::rhaSR-PrhaBAD-mScarlet. Made by mating pLD4358 into LD0.	This study
LD4374	PA14 Δ*gacA* attB::rhaSR-PrhaBAD-mScarlet	PA14 attB::rhaSR-PrhaBAD-mScarlet. Made by mating pLD4358 into LD3679.	This study
LD4376	PA14 Δ*pilA* attB::rhaSR-PrhaBAD-mScarlet	PA14 attB::rhaSR-PrhaBAD-mScarlet. Made by mating pLD4358 into LD3986.	This study
LD4992	PA14 Δ*wbpM* attB::rhaSR-PrhaBAD-mScarlet	PA14 attB::rhaSR-PrhaBAD-mScarlet. Made by mating pLD4358 into LD4635.	This study
LD5052	PA14 Δ*wbpM* Δ*pilA* attB::rhaSR-PrhaBAD-mScarlet	PA14 attB::rhaSR-PrhaBAD-mScarlet. Made by mating pLD4358 into LD4836.	This study
E. coli strains			
LD44	UQ950	E. coli DH5α λ(pir) host for cloning; F-Δ(*argF-lac*)169 Φ80 d*lacZ58*(ΔM15) *glnV44*(AS) *rfbD1 gyrA96*(NalR) *recA1 endA1 spoT1 thi-1 hsdR17 deoR* λpir+	D. Lies
LD661	BW29427	Donor strain for conjugation: *thrB1004 pro thi rpsL hsdS lacZ* ΔM15RP4–1360 Δ(*araBAD*)567 Δ*dapA1341*::[*erm pir*(wt)]	W. Metcalf
LD69	β2155	Helper strain. *thrB1004 pro thi strA hsdsS lacZ*ΔM15 (*F' lacZΔM15* $lacI^q$ *traΔ36* $proA^+$ $proB^+$) Δ*dapA::erm* (Erm^r)*pir::*RP4 [*::kan* (Km^r) from SM10]	[9]
LD2901	S17-1	Str^R, Tp^R, F^- RP4-2-Tc::Mu *aphA*::Tn7 *recA* λpir lysogen	R. Simon
Saccharomyces cerevisiae strains			
LD676	InvSc1	*MATα/MATα leu2/leu2 trp1-289/trp1-289 ura3-52/ura3-52 his3-Δ1/his3-Δ1*	Invitrogen

1. Rahme LG, Stevens EJ, Wolfort SF, Shao J, Tompkins RG, Ausubel FM. Common virulence factors for bacterial pathogenicity in plants and animals. Science. 1995;268: 1899–1902.
2. Recinos DA, Sekedat MD, Hernandez A, Cohen TS, Sakhtah H, Prince AS, et al. Redundant phenazine operons in Pseudomonas aeruginosa exhibit environment-dependent expression and differential roles in pathogenicity. Proc Natl Acad

Sci U S A. 2012;109: 19420–19425.
3. Lin Y-C, Sekedat MD, Cornell WC, Silva GM, Okegbe C, Price-Whelan A, et al. Phenazines regulate Nap-dependent denitrification in Pseudomonas aeruginosa biofilms. J Bacteriol. 2018. doi:10.1128/JB.00031-18
4. Hölscher T, Bartels B, Lin Y-C, Gallegos-Monterrosa R, Price-Whelan A, Kolter R, et al. Motility, Chemotaxis and Aerotaxis Contribute to Competitiveness during Bacterial Pellicle Biofilm Development. J Mol Biol. 2015;427: 3695–3708.
5. Evans CR, Smiley MK, Thio SA, Wei M, Price-Whelan A, Min W, et al. Spatial heterogeneity in biofilm metabolism elicited by local control of phenazine methylation. bioRxiv. 2023. doi:10.1101/2023.02.15.528762
6. Jo J, Cortez KL, Cornell WC, Price-Whelan A, Dietrich LE. An orphan cbb3-type cytochrome oxidase subunit supports Pseudomonas aeruginosa biofilm growth and virulence. Elife. 2017;6. doi:10.7554/eLife.30205
7. Okegbe C, Fields BL, Cole SJ, Beierschmitt C, Morgan CJ, Price-Whelan A, et al. Electron-shuttling antibiotics structure bacterial communities by modulating cellular levels of c-di-GMP. Proc Natl Acad Sci U S A. 2017;114: E5236–E5245.
8. Lin Y-C, Cornell WC, Jo J, Price-Whelan A, Dietrich LEP. The Pseudomonas aeruginosa Complement of Lactate Dehydrogenases Enables Use of d- and l-Lactate and Metabolic Cross-Feeding. MBio. 2018;9. doi:10.1128/mBio.00961-18
9. Dehio C, Meyer M. Maintenance of broad-host-range incompatibility group P and group Q plasmids and transposition of Tn5 in Bartonella henselae following conjugal plasmid transfer from Escherichia coli. J Bacteriol. 1997;179: 538–540.
