# Supplementary material for: Cellular arrangement impacts metabolic activity and antibiotic tolerance in Pseudomonas aeruginosa biofilms
Source: PLoS Biol. 2024 Feb 1;22(2):e3002205. doi: 10.1371/journal.pbio.3002205 (PMC10833521; doi:10.1371/journal.pbio.3002205)
Supplement: S2 Table — (PDF) [file pbio.3002205.s013.pdf]

Table S2. Plasmids used in this study.

Plasmid Name	Description	Source
pMQ30	Yeast-based allelic-exchange vector; *sacB*$^+$, CEN/ARSH, URA3$^+$, Gm^R.	[1]
pFLP2	Site-specific excision vector with cI857-controlled FLP recombinase. encoding sequence, sacB$^+$, Amp^R. Used to insert LD2722-based plasmids into P. aeruginosa strains.	[2]
pLD2722	Gm^R, Tet^R flanked by Flp recombinase target (FRT) sites to resolve out resistance cassettes.	[3]
pLD68	Gm^R, Cm^R mini-Tn7 $P_{A1/04/03}$-*yfp*	[1]
pLD3433	Gm^R, Cm^R mini-Tn7 $P_{A1/04/03}$-*mScarlet*	This study
pLD3655	Gm^R, Cm^R mini-Tn7 $P_{A1/04/03}$-eGFP	This study
pLD4042	Δ*vfr (PA14_08370)* PCR fragment introduced into pMQ30 by gap repair cloning in yeast strain InvSc1.	This study
pLD4537	Δ*antABC (PA14_32160*, *PA14_32150*, and *PA14_32140)* PCR fragment introduced into pMQ30 by gap repair cloning in yeast strain InvSc1.	This study
pLD3079	Δ*gacA (PA14_30650)* PCR fragment introduced into pMQ30 by gap repair cloning in yeast strain InvSc1.	This study
pLD4228	Δ*lasR (PA14_45960)* PCR fragment introduced into pMQ30 by gap repair cloning in yeast strain InvSc1.	This study
pLD3594	Δ*aer1 (PA14_44300)* PCR fragment introduced into pMQ30 by gap repair cloning in yeast strain InvSc1.	This study
pLD3579	Δ*aer2 (PA14_02220)* PCR fragment introduced into pMQ30 by gap repair cloning in yeast strain InvSc1.	This study
pLD3594	Δ*aer1* Δ*aer2 (PA14_44300* and *PA14_02220)* PCR fragment introduced into pMQ30 by gap repair cloning in yeast strain InvSc1.	This study
pLD3979	Δ*pilA (PA14_58730)* PCR fragment introduced into pMQ30 by gap repair cloning in yeast strain InvSc1.	This study
pLD4137	Δ*pilT* Δ*pilU (PA14_05180 and PA14_05190)* PCR fragment introduced into pMQ30 by gap repair cloning in yeast strain InvSc1.	This study
pLD4630	Δ*ssg (PA14_66120)* PCR fragment introduced into pMQ30 by gap repair cloning in yeast strain InvSc1.	This study
pLD4832	Δ*wapR (PA14_66110)* PCR fragment introduced into pMQ30 by gap repair cloning in yeast strain InvSc1.	This study

pLD4631	Δ*wbpM (PA14_23470)* PCR fragment introduced into pMQ30 by gap repair cloning in yeast strain InvSc1.	This study
pLD3939	Δ*cheY (PA14_45620)* PCR fragment introduced into pMQ30 by gap repair cloning in yeast strain InvSc1.	This study
pLD3629	Δ*motA* Δ*motB* (*PA14_65450* and *PA14_65430)* PCR fragment introduced into pMQ30 by gap repair cloning in yeast strain InvSc1.	This study
pLD3630	Δ*motC* Δ*motD (PA14_45560* and *PA14_45540)* PCR fragment introduced into pMQ30 by gap repair cloning in yeast strain InvSc1.	This study
pLD3938	Δ*fliA (PA14_45630)* PCR fragment introduced into pMQ30 by gap repair cloning in yeast strain InvSc1.	This study
pLD3635	Δ*ptsP (PA14_04410)* PCR fragment introduced into pMQ30 by gap repair cloning in yeast strain InvSc1.	This study
pLD1204	Δ*dipA (PA14_66320)* PCR fragment introduced into pMQ30 by gap repair cloning in yeast strain InvSc1.	This study
pLD3910	Δ*cyaA PA14_69610* PCR fragment introduced into pMQ30 by gap repair cloning in yeast strain InvSc1.	This study
pLD3909	Δ*cpdA (PA14_65690)* PCR fragment introduced into pMQ30 by gap repair cloning in yeast strain InvSc1.	This study
pLD3609	Δ*ackA (PA14_53470)* PCR fragment introduced into pMQ30 by gap repair cloning in yeast strain InvSc1.	This study
pJM260	miniCTX1-rhaSR-PrhaBAD-stRBS-aacC1	[4]
pLD3208	MCS-mScarlet GmR, TetR flanked by Flp recombinase target (FRT) sites to resolve out resistance cassettes. Cloned by swapping gfp sequence with mScarlet (XhoI + SacI).	[5]
pLD4358	Gm^R, Cm^R mini-Tn7 $P_{A1/04/03}$*::rhaSR-PrhaBAD mScarlet.* The *rhaSR-PrhaBAD* promoter was amplified from pJM260 using primers LD3920 and LD3920 and inserted into pLD3208 using SpeI and XhoI.	This study

1. Shanks RMQ, Caiazza NC, Hinsa SM, Toutain CM, O'Toole GA. Saccharomyces cerevisiae-based molecular tool kit for manipulation of genes from gram-negative bacteria. Appl Environ Microbiol. 2006;72: 5027–5036.
2. Hoang TT, Karkhoff-Schweizer RR, Kutchma AJ, Schweizer HP. A broad-host-range Flp-FRT recombination system for site-specific excision of chromosomally-located DNA sequences: application for isolation of unmarked Pseudomonas aeruginosa mutants. Gene. 1998;212: 77–86.
3. Jo J, Cortez KL, Cornell WC, Price-Whelan A, Dietrich LE. An orphan cbb3-type cytochrome oxidase subunit supports Pseudomonas aeruginosa biofilm growth and virulence. Elife. 2017;6. doi:10.7554/eLife.30205
4. Meisner J, Goldberg JB. The Escherichia coli rhaSR-PrhaBAD Inducible Promoter System Allows Tightly Controlled Gene Expression over a Wide Range in Pseudomonas aeruginosa. Appl Environ Microbiol. 2016;82: 6715–6727.
5. Wang B, Lin Y-C, Vasquez-Rifo A, Jo J, Price-Whelan A, McDonald ST, et al. Pseudomonas aeruginosa PA14 produces R-bodies, extendable protein polymers with roles in host colonization and virulence. Nat Commun. 2021;12: 1–14.
