# Supplementary material for: Cellular arrangement impacts metabolic activity and antibiotic tolerance in Pseudomonas aeruginosa biofilms
Source: PLoS Biol. 2024 Feb 1;22(2):e3002205. doi: 10.1371/journal.pbio.3002205 (PMC10833521; doi:10.1371/journal.pbio.3002205)
Supplement: S3 Table — (PDF) [file pbio.3002205.s014.pdf]

Primer	Sequence
Primers for plasmid pLD4042 (used to make Δ*vfr*)	
3611	cctgcaggtcgactctagagGGGAGAAGATGGACGAACTG
3612	CTACACCGCAAAGAGCACCCTGGTGCATGTGAAAGGAAA
3613	TTTCCTTTCACATGCACCAGGGTGCTCTTTGCGGTGTAG
3614	cagctatgaccatgattacg GATGGCGATTGTGGTTTGTT
Primers for plasmid pLD4537 (used to make Δ*antABC*)	
3962	ggcaaattctgttttatcagaccgcttctgcgttctgatTCAGTCCCTTGATCAGCGC
3963	GTCGAAATGCTCGTGCAGGTACAGCTCCGGTTCGGTGAAC
3964	GTTCACCGAACCGGAGCTGTACCTGCACGAGCATTTCGAC
3965	aattgtgagcggataacaatttcacacaggaaacagctCCCGGCGAATTTCTACTACGA
Primers for plasmid pLD3079 (used to make Δ*gacA*)	
1932	ccaggcaaattctgttttatcagaccgcttctgcgttctgatAGCGTGCGACGTTATGTCT
1933	gaagatgcggtagcgatagACCACTTGCAAGCCTTCG
1934	cgaaggcttgcaagtggtCTATCGCTACCGCATCTTC
1935	ggaattgtgagcggataacaatttcacacaggaaacagctTCACGTTGACGATCAACTCC
Primers for plasmid pLD4228 (used to make Δ*lasR*)	
3813	cctgcaggtcgactctagagACAGGTCCCCGTCATGAAAC
3814	GTTTTCTTGAGCTGGAACGCATGGCCGTTAATTTGGGTCT
3815	AGACCCAAATTAACGGCCATGCGTTCCAGCTCAAGAAAAC
3816	cagctatgaccatgattacgGATCAACATGGTCACCTCCA
Primers for plasmid pLD3594 (used to make Δ*aer1*)	
2958	aggcaaattctgttttatcagaccgcttctgcgttctgatTGGAACCGAAGGACTTCAAC
2942	CTCGTCGGAGGTCTGCTCTTTGAGATTGGTGGTGGTGA
2943	TCACCACCACCAATCTCAAAGAGCAGACCTCCGACGAG
2944	ggaattgtgagcggataacaatttcacacaggaaacagctAGATCCTCTGCGGCGATT
Primers for plasmid pLD3579 (used to make Δ*aer2*)	
2950	aggcaaattctgttttatcagaccgcttctgcgttctgatCACGGTGACGATCACCAGGT
2951	CTCCAGTCGTTCGCCGACAGCGTGGTCCAGCTCGCC
2952	GGCGAGCTGGACCACGCTGTCGGCGAACGACTGGAG
2953	ggaattgtgagcggataacaatttcacacaggaaacagctCTGGCAACTGGTGTTCATGC
Primers for plasmid pLD3979 (used to make Δ*pilA*)	
3583	aggcaaattctgttttatcagaccgcttctgcgttctgatCCTCACCCTCTGAACGAATC
3584	GATTACACCATTCGTGCTCG ATCCAATGCGGTTTCCAGT
3585	ACTGGAAACCGCATTGGAT CGAGCACGAATGGTGTAATC
3586	ggaattgtgagcggataacaatttcacacaggaaacagctACTTCATGGAGCATGCCATC

Primers for plasmid pLD4137 (used to make Δ*pilT* Δ*pilU*)	
3666	aggcaaattctgttttatcagaccgcttctgcgttctgatGAACGCTATGCGGGGTGT
3667	GTCAGGTTCGACAGGTGGTC GGAGAGGTGCAGGTCCGAAG
3668	CTTCGGACCTGCACCTCTCC GACCACCTGTCGAACCTGAC
3669	ggaattgtgagcggataacaatttcacacaggaaacagctGCCTATCCTGTAGGCTGGTG
Primers for plasmid pLD4630 (used to make Δ*ssg*)	
4193	aggcaaattctgttttatcagaccgcttctgcgttctgatAGAAAAACAACCGCGCAAT
4194	GGCTACTTCCGCAAGCACGCCGCTGTCGGTCAGAGTATC
4195	GATACTCTGACCGACAGCGGCGTGCTTGCGGAAGTAGCC
4196	ggaattgtgagcggataacaatttcacacaggaaacagctTACCTGAACCTGACGGGACT
Primers for plasmid pLD4832 (used to make Δ*wapR*)	
4349	aggcaaattctgttttatcagaccgcttctgcgttctgatGGCATGATCGTGGCTTATATC
4350	CAGACGTACCGCAACGTCTCGAACCGGGACAAGGT
4351	ACCTTGTCCCGGTTCGAGACGTTGCGGTACGTCTG
4352	ggaattgtgagcggataacaatttcacacaggaaacagctAAGAATTTCGAGGCGATGG
Primers for plasmid pLD4631 (used to make Δ*wbpM*)	
4201	aggcaaattctgttttatcagaccgcttctgcgttctgatCATGGATGGCATTGATGGTA
4202	GTAGTCGTCCTTCTCCACGGCGAAGGCTAGCCAGAGCGATA
4203	TATCGCTCTGGCTAGCCTTCGCCGTGGAGAAGGACGACTAC
4204	ggaattgtgagcggataacaatttcacacaggaaacagctGGTGATCCGCACCAACTATT
Primers for plasmid pLD3939 (used to make Δ*cheY*)	
3424	aggcaaattctgttttatcagaccgcttctgcgttctgat CCCAGAGACGTGTTACTCGAA
3425	TCAACGGCTATGTGGTCAAAGGACTTGGGCTTCACCAATA
3426	TATTGGTGAAGCCCAAGTCCTCAACGGCTATGTGGTCAAA
3427	ggaattgtgagcggataacaatttcacacaggaaacagctGAGCCGTATACGAGGCATTC
Primers for plasmid pLD3629 (used to make Δ*motA* Δ*motB*)	
2994	aggcaaattctgttttatcagaccgcttctgcgttctgatGCATCGAAAAGATCCAGGAA
2995	ATCCAGCCCTTCGAGGTCGTGCAGAAGAAGCTCAACC
2996	GGTTGAGCTTCTTCTGCACGACCTCGAAGGGCTGGAT
2997	ggaattgtgagcggataacaatttcacacaggaaacagctACCTGCTGCTGGACTTCG
Primers for plasmid pLD3630 (used to make Δ*motC* Δ*motD*)	
3002	aggcaaattctgttttatcagaccgcttctgcgttctgatGTAGGACTGCAAGGCCAGTT
3003	ATCATCCTGGCGTTCGTCGGCACGCAAGCTGCAGATACT
3004	AGTATCTGCAGCTTGCGTGCCGACGAACGCCAGGATGAT
3005	ggaattgtgagcggataacaatttcacacaggaaacagctCCGGTCCTGATGTTCTCCT

Primers for plasmid pLD3938 (used to make Δ*fliA*)	
3484	aggcaaattctgttttatcagaccgcttctgcgttctgatGAACTGTTCGATGCGCTTG
3485	CATCGCCTATCACCTGCTCGAGGAGATCGGCGAGGTACTG
3486	CAGTACCTCGCCGATCTCCTCGAGCAGGTGATAGGCGATG
3487	ggaattgtgagcggataacaatttcacacaggaaacagctCATCGTTCCCGCCAATAC
Primers for plasmid pLD3635 (used to make Δ*ptsP*)	
2475	aggcaaattctgttttatcagaccgcttctgcgttctgatCGGCTCGATCACTTCCTGCA
2476	ctgcgggttgtcgaaggtgagCATGGCTTCCTTGACCCGCTG
2477	cagcgggtcaaggaagccatgCTCACCTTCGACAACCCGCAG
2478	ggaattgtgagcggataacaatttcacacaggaaacagctGATGAACTCCTCGCCGCCC
Primers for plasmid pLD1204 (used to make Δ*dipA*)	
2611	ggaattgtgagcggataacaatttcacacaggaaacagctGCAGCAACTGGTGAAGCGC
2612	GCAGCTCGCTGAGGTTCTcgcGCGCCGGAAGCTCGGA
2613	TCCGAGCTTCCGGCGCgcgAGAACCTCAGCGAGCTGCT
2614	ccaggcaaattctgttttatcagaccgcttctgcgttctgatATCTGCAGCTCGCCCAG
Primers for plasmid pLD3910 (used to make Δ*cyaA*)	
3502	cctgcaggtcgactctagagCAGCTCAATCCCTACCCCTA
3503	TAACGCAGATAGTGCAGCGTGTCGAGATCGAGGCTGAGTG
3504	CACTCAGCCTCGATCTCGACACGCTGCACTATCTGCGTTA
3505	cagctatgaccatgattacgAAGGCAAGGTCTCGATCCTC
Primers for plasmid pLD3909 (used to make Δ*cpdA*)	
3494	cctgcaggtcgactctagagGTCGATGACTTCCAGCGAGT
3495	CGTACTGCTGGTGCAGCTTTTCGAAGTGGACTACGACACC
3496	GGTGTCGTAGTCCACTTCGAAAAGCTGCACCAGCAGTACG
3497	cagctatgaccatgattacg CCTGATCGACAAGGACGAG
Primers for plasmid pLD3609 (used to make Δ*ackA*)	
3022	caggcaaattctgttttatcagaccgcttctgcgttctgatCAATCGGCGAATGAACGCCG
3023	GGATCACCAGTACCCGCGGGTGCAGGGGAAACAGGGAGT
3024	ACTCCCTGTTTCCCCTGCACCCGCGGGTACTGGTGATCC
3025	ggaattgtgagcggataacaatttcacacaggaaacagctCGTGCGCTGAGCAGATCG
Primers for plasmid pLD4358 (used to make pSEK109-rhaSR-PrhaBAD)	
3920	gattcgactgcactagtTTAATCTTTCTGCGAATTGAGATGAC
3921	gattcgactgcctcgagTACGACCAGTCTAAAAAGCGC
