# Supplementary material for: Cellular arrangement impacts metabolic activity and antibiotic tolerance in Pseudomonas aeruginosa biofilms
Source: PLoS Biol. 2024 Feb 1;22(2):e3002205. doi: 10.1371/journal.pbio.3002205 (PMC10833521; doi:10.1371/journal.pbio.3002205)
Supplement: Attachment — Submitted filename: Response to reviewers.pdf [file pbio.3002205.s017.pdf]

November 14, 2023

Editorial Office

PLoS Biology

Re: Resubmission of manuscript (PBIOLOGY-D-23-01462R1) - [EMID:996f28b86de10f2d]

Dear Dr. Jauregui, dear reviewers,

Thank you for considering our manuscript, entitled "**Cell arrangement impacts metabolic activity and antibiotic tolerance in *Pseudomonas aeruginosa* biofilms**", which was submitted in June. My co-authors and I very much appreciate the reviewers' constructive feedback and the editors' invitation to submit a revised manuscript.

The current manuscript contains new experimental results including quantification of labeled cells in mature biofilms (Fig. S1), quantification of D_2O distribution in biofilms (Fig. S2), visualization of cellular arrangement in anoxic conditions (Fig. S3), imaging of macrocolony biofilm morphology (Fig. S4), quantification of RhaSR-PrhaBAD-controlled mScarlet expression in liquid culture (Fig. S8), visualization and quantification of dye distribution in pellicle biofilms (Fig. S9), and metabolic activity quantification for a Δ*ssg* biofilm (Fig. S10). We have revised the text to provide context for and interpretation of these results, and we feel that these changes have greatly enhanced the paper.

We have compiled a point-by-point response to reviewers' comments, pasted below. Our responses are in blue.

Thank you very much for your time.

Sincerely,

Lars Dietrich

—-----------------------

Reviewer #1:

In the manuscript "Cell arrangement impacts metabolic activity and antibiotic tolerance in Pseudomonas aeruginosa biofilms," Dayton et al. conducted a detailed investigation into the cellular organization within P. aeruginosa colony biofilms. The authors explored the role of various cell factors, including adhesins, sigma factors, second messenger signalling factors, and metabolic factors, in determining a distinctive pattern of cellular arrangement. They also examined how this pattern influenced the metabolic activity and survival capacity of cells when exposed to antibiotics. Specifically, the authors found that in an internal zone (zone 2) cells form long vertically oriented chains referred to as "striations" and identified cell surface components (pilus and O-antigen) that impacted such pattern of cellular organization across the biofilm depth. Using stimulated Raman scattering microscopy and dead cell labelling, the authors described relationships between cellular arrangement, cell metabolic status, and susceptibility to antibiotic. Overall, it is very nice study that provides novel insights into an aspect of bacterial biofilms that has not been extensively explored, which is the role of cellular organization in biofilm physiology. I think this work can be of great interest for researchers in cell biology and,

particularly, for the biofilm researcher community. The study is well-conducted, with robust data and proper statistical analysis. I have made some comments, which I present below.

Thank you for your positive comments on our work!

Comments:

- The approach used to visualize "striations" in biofilms involved inoculating 2.5% cells that constitutively express mScarlet, representing a 1:40 ratio of mScarlet-labelled cells to non-labelled cells. Upon reviewing the images in the various figures, it appears that the number of mScarlet-expressing cells is higher than the initial 1:40 ratio. Are mScarlet-labelled cells growing faster or out competing non-labelled cells?

Thank you for this observation. We agree that the ratio of mScarlet-labeled to -unlabeled cells appears to exceed the initial ratio of 1:40. Because our biofilm sections were 5-μm thick, each section image shows the integrated fluorescence signal of 5-10 cell layers (given that one cell is 0.5 to 1 μm thick). This may have led to the apparent higher ratio of mScarlet-labeled cells. To address this possibility experimentally, we homogenized 3-day-old biofilms and counted fluorescently labeled and unlabeled cells. We found that, indeed, only ~2-3% of cells were labeled. This quantification is now shown in Figure S1. We also now indicate in the Methods section that each section was 10-μm thick and that the image shown is a composite (“Z-stack”) of images taken at 5 focal planes at 1-μm intervals

Early in the Result/Discussion section the authors presented data on how the availability of resources (tryptone concentration) affects the organization of cellular-arrangement zones. Later in the same section, they presented data on how cell patterning influences the distribution of substrates (rhamnose and microspheres). Although these subsections appear to be related, the use of different substrates (tryptone vs rhamnose) and terminology (resources vs substrates) creates confusion. The authors should clarify the relationship between these findings and consider revising the text to ensure a more cohesive presentation.

Thank you for this feedback. We agree that the indicated distinctions and precise terminology are important. We now define these terms in lines 56-58. In our view, the subsections regarding (i) the effects of tryptone concentration and (ii) the distribution of rhamnose and microspheres address different aspects of the structural biology of the biofilm. Experiments described in the first subsection test whether absolute resource (tryptone) availability affects biofilm organization, while those in the second subsection test whether the distribution of an added molecule (rhamnose) or particle (fluorescent bead) are affected by cellular arrangement in biofilms grown under standard conditions of resource (tryptone) availability. To clarify this, we now use the terms “molecule” and “particle” to refer to rhamnose and fluorescent beads, respectively.

We sought to test the hypothesis that cellular arrangement affects the distribution of molecules and particles in biofilms and chose rhamnose and fluorescent beads because they are drastically different in size. The biofilms were grown under high-resource conditions (i.e., on

trytpone medium), and in this context, rhamnose acts primarily as a cue to activate expression of the PrhaBAD system; the fluorescence produced by this system therefore serves to report the distribution of rhamnose. Nevertheless, we acknowledge the caveat that *P. aeruginosa* may be able to metabolize rhamnose and in light of this, we have now performed additional experiments examining the effect of cell arrangement on the distribution of an inert dye. The results of these experiments are shown in Figure S9 and discussed in lines 389-393.

Related to the data presented in the subsection "Cell patterning influences the distribution of substrates across biofilm depth", I am not sure if the L-rhamnose reporter system and the microspheres are the appropriate systems to evaluate the distribution of substrates across the biofilm. In the case of the rhaSRPrhaBAD inducible promoter system, the authors already indicated that pleiotropic effects inhibited activity of the system in biofilm zones 1 and 2. In the case of the microspheres, given their size I would not consider it as a diffusible substrate. The fact that beads remain at the agar interface in the pilA mutant biofilm (Fig 6H), indeed reflects that beads are somehow limited to enter into the biofilm, but this does not necessarily imply an inability of diffusible substrates to penetrate or distribute differentially. Have the authors considered alternative approaches or additional experiments for a more accurate assessment of substrate distribution within the biofilm?

As discussed above, our intention in choosing rhamnose and microspheres was to probe whether structural differences between wild-type and mutant biofilms affect the distribution of molecules and particles of different sizes. We used rhamnose as a proxy for small diffusible substrates and beads as a proxy for a larger particle. We feel that the ability of larger particles to enter a biofilm is worth testing because it might impact biofilm composition in terrestrial or aquatic settings by modulating uptake of abiotic particles or other microbial species. Because activity of the rhamnose-inducible reporter depends not only on the distribution of rhamnose within the biofilm, but also on its uptake into cells and expression of the fluorescent reporter, we have now conducted additional experiments with an inert fluorescent dye and results are included in Figure S9.

Based on the data presented in Figure 7, the authors' interpretation regarding the correlation between cellular arrangement and antibiotic tolerance is understandable. However, it is evident that the zone exhibiting vertical striations is significantly thicker (about 130 μm) than the zone of antibiotic susceptibility (20-60 μm). Although the subzone ranging from 60 to 130 μm displays the same cellular organization pattern as the 20-60 μm subzone, cells are only susceptible within the latter, where high metabolic activity occurs due to optimized oxygen and nutrient availability. It appears that the ordered cells in the 60-130 μm subzone do not prevent the antibiotic from reaching cells above that subzone. Thus, the relationship presented may not be as direct as suggested.

Thank you for bringing this up. We agree that the zone of metabolic activity does not appear to solely be determined by the zone of striations, although it is interesting that it does correlate with the striated zone, even when the location of this zone changes due to resource/nutrient limitation as shown in Figure 2. Our interpretation of the results of the antibiotic-treatment

experiments is that a combination of the location of high metabolic activity and cellular arrangement affects the zone of antibiotic susceptibility (discussed in lines 455-489).

The manuscript is well-written and structured. However, while the "Results and Discussion" section adequately describes the results and their corresponding conclusions, it would benefit from a more extensive discussion. Although some discussions of the results are provided, the authors should consider expanding on certain aspects not discussed.

We appreciate your feedback on the content of the Results and Discussion section. We have increased the amount of discussion by adding text, as suggested by each of the reviewers, and citing relevant work on the following topics:
-entry into, and uptake of, molecules and particles into biofilms
-role of metabolic activity in determining antibiotic susceptibility and the colocalization of these properties in biofilms
-specific examples of ordered cellular arrangement observed in diverse bacterial culturing systems and species
-the prior observation that mutants defective in O-antigen attachment show altered cellular aggregation phenotypes in an in vitro cystic fibrosis model

Reviewer #2:
This is an exceptional work from the Dietrich lab that demonstrates microanatomy of Pseudomonas aeruginosa colony biofilm and highlights parallel arranged cells that influence metabolism and therefore also specific (grown-dependent) antibiotic sensitivity. The work includes molecular details (mutants) and elegantly performed microscopy approaches. I have only minor comments that should be easily addressed.

Thank you for the positive feedback!

Technical point: The authors should explain if mScarlet overexpression has any influence on the fitness of the cells. This might have been described previously or tested by the authors to circumvent any bias caused by the fluorescent marker. When mixing 2.5% of fluorescent marker labeled strains, will the 2.5% roughly remains at the end of the experiments? Such quantification of strain abundance after growth might answer this concern.

Thank you for bringing this up. We agree that it is important to test whether the fluorescent marker affected fitness, and we have now done this by quantifying colony-forming units form a 3-day-old biofilm. The results, included as Figure S1, confirm that the initial inoculation ratio of 1:40 (mScarlet-labeled to unlabeled cells) is still present at the end of the incubation period.

The manuscript is written by a direct style, which I let the editor to decide whether it should be changed or remain as it is, e.g. line 160 "we were surprised", line 320: "we will therefore focus our interpretation"

We like a direct writing style because we feel it makes the results more accessible to the reader, but will defer to the editor regarding stylistic changes. Regarding the two instances mentioned, we would like to retain the phrase “we were surprised” because it refers to a totally unexpected result and we think one instance of the word “surprised” is acceptable in a manuscript ;) However, as suggested, we have revised the phrase “we will therefore focus our interpretation”.

Line 182: this should be the same for pure cultures also, not only with mixing-assay biofilms, right?

Yes, this is correct. We have modified the sentence accordingly (now line 207).

Line 210: it would benefit the readers if the authors would include in the table which mutation influence colony structure or other type of Pseudomonas biofilms, so it provides a good comparison to the microanatomy. The authors conclude that macroscale does not correlate with cellular arrangement, but this is hard to evaluate without knowing the full literature. Adding that background information in the table would make it easier to evaluate.

Thank you, we agree that this information will be helpful to the reader. Because the results of the colony morphology assay have not been published for several of the mutants listed, we revised the text (rather than the table) and have included references for various mutants that have either WT or altered colony biofilm morphotypes (lines 276-292). We have also added a figure showing the colony morphology phenotypes for the main mutants of interest in the paper (now provided as Figure S4). Together, these revisions and additions are sufficient to support the point that changes in macroscale biofilm structure do not correlate with changes in biofilm microanatomy.

Line 229: Is wbpM known to be regulated by GacS/GacA or LasR system? Does this gene display different transcript level in these mutants in previous (biofilm) transcriptomes preformed with gacS/gacA or lasR mutants?

We appreciated this potential connection, but in reviewing the literature we did not find evidence that wbpM is regulated by the GacS/GacA or LasR system.

Scale bar should be added to the CLSM images (mixing assays) in Fig 1B and C (I understand that orientation panels practically include the scale, but in later figures, that is not added); Fig 2B, Fig 3B Fig 4B, Fig 5C, Fig 7D

Thank you for pointing this out. Scale bars and related information have been added to all figures and figure legends.

Superb work, congratulations!
ATK

Reviewer #3:

In this manuscript, the authors investigate the effect of different vertical cellular arrangements in a colony on agar. The found that depending upon the region of the colony, different arrangements are observed. They identified type IV pili mutants as defective for normal cellular arrangements in colonies. In general, this is an interesting manuscript with some cool observations. But little is provided mechanistically to explain many of the observations that have been made, reducing my enthusiasm for the study.

Thank you for your positive comments and constructive criticism on our manuscript. Our goals for this manuscript have been to introduce the fundamental observation of vertically arranged clonal striations in *P. aeruginosa* biofilms, identify genetic determinants of the cellular arrangement, and describe correlations between cellular arrangement and other physical and physiological properties of these biofilms. Our discovery that functional pili and attached O-antigen are required for wild-type biofilm microanatomy hints at mechanisms that influence development at the cellular scale, and we have added text to the Results and Discussion section that describes these mechanisms.

The introduction is unusually vague and brief. I would restructure it to inform the readers about what is known regarding biofilm structure and the environmental factors that influence it, paying attention to the many excellent papers measuring vertical nutrient gradients in P. aeruginosa colony biofilms (PS Stewart and others).

We have revised the introduction and results/discussion sections to provide additional information regarding the structural development of, and gradient formation in, biofilms, tailoring our references to cover the work of Stewart et al, Drescher et al., and others (lines 64-70 and 157-161). We have also added detail on the topic of how biofilm anatomy influences the uptake and distribution of substrates and how this relates to antimicrobial susceptibility, again referencing the work of these authors and others (lines 65-73 and 214-217).

Related to the data shown in figure 2. The inverse gradients of O2 (High to low from colony top to bottom) and nutrients from the agar (high to low from colony bottom to top) make the data presented here hard to interpret. I think the authors should grow their colony biofilms anaerobically on agar containing nitrate, and see if the trends they have observed hold up. By using nitrate anaerobically, the key gradients would be aligned vertically from colony bottom to top (for terminal electron acceptor and nutrients).

We were also very interested to know whether an alternate electron acceptor at the biofilm base would affect striation formation. To address this, we grew colony biofilms on agar medium containing potassium nitrate under both oxic and anoxic conditions. Biofilms grown anaerobically on nitrate did not form striations, indicating that the oxygen gradient is crucial to their formation. In contrast, biofilms grown aerobically on medium containing nitrate formed striations that were similar to those formed in the absence of added nitrate–indicating that nitrate addition per se does not affect striation formation. These results are now included in Figure S3 and described in lines 221-227.

I think it is very important for the authors to demonstrate that there is not a gradient of D2O availability vertically across the colonies. D2O bioavailability in different regions of the colony could skew the signals observed in Fig 2B.

We agree that this is an important control. We have now quantified the distribution of D2O in a biofilm transferred to D2O-containing medium, via stimulated Raman scattering microscopy and optical sectioning (our conventional fixation/sectioning method was not applicable because we cannot fix water). We found an even distribution of D2O throughout the biofilm. The results are shown Figure S2 and are discussed in lines 211-213.

Sentence lines 216-219 is unclear and should be reworded.

We have reworded this sentence to improve clarity (now lines 243-246).

What are the metabolic activity gradients for the different mutant arrangements shown in Fig 3?

The metabolic activities of the mutants, except for Δ*ssg*, were shown in Figure 7B. We have added the metabolic activity quantification for Δ*ssg* in Figure S10.

Line 248. Fig 3D??

This typo has been corrected to say 3B.

Lines 276-278 Reports from the Diggle/Goldberg labs have already shown that LPS alterations can influence cellular distribution and packing.

Yes. We had commented on their findings and cited their work, but we now also mention their findings at an earlier point in the manuscript in lines 267-269 (the new location is line 354-357) and appears later due to the additional text we have added across the revised manuscript).

The major approach that supports most of the authors conclusions are based on microscopy applied to "mixing" experiments. It would be much more convincing if there were additional lines of experimentation or methodologies to support and complement the mixing experiments.

In the original manuscript we described the use of several different approaches to analyze the microanatomies, and the physiological effects of altered microanatomy, of macrocolony and pellicle biofilms, including (i) sample imaging by scanning electron microscopy (Figure 1A), (ii) characterizing the uptake and distribution of an added chemical cue (rhamnose) and fluorescent beads (Figure 6), and (iii) imaging of metabolic activity via stimulated Raman scattering microscopy (Figures 2B, 7B, and S10) in addition to (iv) fluorescence imaging of a labeled subpopulation (the “mixing” assay) (Figure 1B).

Independently, we have observed that a stochastically expressed native promoter, when cloned in front of the mScarlet gene, also promotes the formation of fluorescent striations in *P.*

aeruginosa macrocolony biofilms (Wang et al. 2021) (now discussed in lines 138-141). We have also independently developed a method for imaging of developing pellicle biofilms that visualizes the formation of striations in real time (Chen et al. 2023). Each of these points has been added to the text.

Finally, the revised manuscript now also includes results from an additional approach in which we monitored the uptake and distribution of a fluorescent dye in pellicle biofilms (Figure S9).

We feel that together, the combined use of these various approaches and references to independent observations provide a characterization of *P. aeruginosa* biofilm microanatomy from diverse angles.

References:

Wang B, Lin YC, Vasquez-Rifo A, Jo J, Price-Whelan A, McDonald ST, Brown LM, Sieben C, Dietrich LEP. Pseudomonas aeruginosa PA14 produces R-bodies, extendable protein polymers with roles in host colonization and virulence. Nat Commun. 2021 Jul 29;12(1):4613. doi: 10.1038/s41467-021-24796-0.

Chen Y, Chauhan S, Gong C, Dayton H, Xu C, Cruz ED, Datta MS, Leong KW, Dietrich LEP, Tomer R. Scalable projected Light Sheet Microscopy for high-resolution imaging of living and cleared samples. bioRxiv [Preprint]. 2023 Jul 18:2023.05.31.543173. doi: 10.1101/2023.05.31.543173.

For the rhamnose-inducible mScarlet, the authors should show in liquid culture that the mutants have the same rhamnose-induced induction curves- a minor point, but a good control experiment.

We agree that this is a good control. We grew the WT and mutants in liquid culture with and without added rhamnose and saw no differences between strains with respect to fluorescence. Results are shown in Figure S8.

The observation that PilA is required for cellular arrangements of the O-antigen mutants is interesting (Fig 5). However, there is no convincing mechanistic explanation for this, making these data very phenomenological.

We agree that this is an interesting finding. In lines 330-333 we speculate that these cell surface components may be linked at the regulatory level or interact directly. In Figure 5B and 5C we quantified PilA protein levels in O-antigen synthesis/attachment mutants and examined cell arrangement in double mutants. We found that total PilA levels do not account for the cellular arrangement phenotype of these mutants and that in the absence of O-antigen-modified LPS, PilA also appears to promote random cellular orientation (now described in lines 335-340 and 342-352). In lines 354-357 we speculate that this could be due to pilus glycosylation, a modification that has been reported for some strains of *P. aeruginosa* (Asikyan et al. 2008).

Reference:
Asikyan ML, Kus JV, Burrows LL. Novel proteins that modulate type IV pilus retraction dynamics in Pseudomonas aeruginosa. J Bacteriol. 2008 Nov;190(21):7022–7034. PMCID: PMC2580705

Fig 6B- its not clear to me why there are vertical striations in some of the MScarlet expression profiles !?

Because zones at distinct distances from the biofilm-agar interface displayed clear differences in cellular arrangement, we had focused our discussion on the overall levels of mScarlet production across depth. But we are also intrigued by these vertically oriented clusters of cells, showing high levels of mScarlet expression. One possibility is that these clusters are adjacent to channels/gaps in the biofilm (which are better penetrated by molecules from the medium) and that they therefore have better access to rhamnose. We have added this point to the text.

The data presented in Fig. 6 F-H are pretty cool, with the pilA mutations affecting microsphere distribution, but the mechanism responsible for this is unclear and the authors suggestion as to why vague. Im not sure at all what this result means.

We had predicted that cellular arrangement would affect the movement of molecules and particles through biofilms because cells in the striated region of WT biofilms show tight, lengthwise packing and cells in the disordered region are randomly arranged with large intercellular gaps (Figure 1). Naturally occurring biofilms are exposed to sources of molecules and particles in a range of sizes and the abilities of these to enter the biofilm structure can affect biofilm physiology and development (Flemming et al. 2016); we therefore sought to test whether mutants with altered arrangement showed differences in the uptake and distribution of molecules and particles. The fact that the Δ*pilA* mutant is defective in microsphere uptake and distribution suggests that cells are more tightly packed in the biofilm and that this inhibits the movement of the particles. We have added this explanation to the text in lines 407-409.

Reference:
Flemming HC, Wingender J, Szewzyk U, Steinberg P, Rice SA, Kjelleberg S. Biofilms: an emergent form of bacterial life. Nat Rev Microbiol. 2016 Aug 11;14(9):563-75. doi: 10.1038/nrmicro.2016.94. PMID: 27510863.

The data shown in Fig 7D have been demonstrated previously by a number of groups- zones of metabolic activity correspond to zones on antimicrobial sensitivity.

Thank you; we appreciate your point that the relationship between metabolic activity and antimicrobial sensitivity has been addressed in the literature and we have revised the text in lines 81-86, 442-451, and 455-457 to include additional references that cover this topic. In the current manuscript, our intention has been to build on prior findings by examining this relationship in the context of biofilm microanatomy and to exploit a new technique, stimulated Raman scattering microscopy, for this purpose. Because our results suggest that differences in cellular arrangement affect the distribution of small molecules in the biofilm, we hypothesized that the zone of metabolic activity would change (due to altered electron donor availability) and that this would correlate with a change in antibiotic susceptibility. We intentionally used tobramycin for these experiments because its uptake requires a proton motive force (the

maintenance of which is enabled by respiration, i.e. the simultaneous availability of electron donors and oxygen) (Meylan et al. 2017).

Reference:
Meylan S, Porter CBM, Yang JH, Belenky P, Gutierrez A, Lobritz MA, Park J, Kim SH, Moskowitz SM, Collins JJ. Carbon Sources Tune Antibiotic Susceptibility in Pseudomonas aeruginosa via Tricarboxylic Acid Cycle Control. Cell Chem Biol. 2017 Feb 16;24(2):195-206. doi: 10.1016/j.chembiol.2016.12.015.

Why is a wbpM mutant showing uniform PI- staining in the colony!?

With respect to PI staining, the Δ*wbpM* mutant shows two changes relative to the WT: (i) an overall increase in staining of untreated biofilms, and (ii) in tobramycin-treated biofilms, a shift toward the biofilm-air interface for (and a slight broadening of) the zone of maximum staining. Effect (i) indicates that the *wbpM* mutation has a more general negative effect on survival in biofilms. However, the shift in the zone of maximum staining for treated biofilms formed by this mutant (effect (ii)) is consistent with their upward shift in the zone of maximum metabolic activity (Figure 7A) and their increased response to rhamnose at the biofilm-air interface (Figure 6B). We have added revised text in lines 459-463 to better describe this finding. Together, these results suggest that the effect of *wbpM* deletion on cellular arrangement and therefore distribution of molecules (which would include cues, nutrients, and antibiotics provided in the agar) to the biofilm-air interface increases susceptibility to antibiotic in this zone.
