# Supplementary material for: Cellular arrangement impacts metabolic activity and antibiotic tolerance in Pseudomonas aeruginosa biofilms
Source: PLoS Biol. 2024 Feb 1;22(2):e3002205. doi: 10.1371/journal.pbio.3002205 (PMC10833521; doi:10.1371/journal.pbio.3002205)
Supplement: Attachment — Submitted filename: Response to editorial comments.pdf [file pbio.3002205.s018.pdf]

December 16, 2023
Editorial Office
PLoS Biology

Re: Resubmission of manuscript (PBIOLOGY-D-23-01462R2) - [EMID:953230555ebbd75e]

Dear Dr. Smith and team,

Thank you for your careful assessment of our manuscript. We have addressed your comments. Our answers are in blue text.

1) TITLE: After some discussion within the team, we think the title might be clearer if changed to "Cellular arrangement impacts metabolic activity and antibiotic tolerance in Pseudomonas aeruginosa biofilms". If you agree, please change it accordingly.

We have changed the title as suggested.

2) Thank you for providing the underlying data related to your figures as a supplemental excel file. A couple of requests:

a. please reference this file in every relevant figure legend (for example, you can add the sentence "the data underlying this figure can be found in S1_raw_data")

Done.

b. I noticed that this file seems to be missing some relevant data. Can you update it to include the raw data related to Figure S1? Also, please add data from orientation plots to this file (ex related to data presented in figures 1B-C; 2B; Fig 3B; Fig 4B; Fig 5B)

We have added the indicated data to the raw data file.

3) METHODS: I noticed that some of the methods are presented as a supplementary file. Can you please move those details into the main text of the manuscript along with the rest of the methods?

We have now integrated the SI Methods with Methods in the main text.

The reference list remained unchanged.

Thank you and best wishes,
Lars

Lars Dietrich
Associate Professor
Biological Sciences
Columbia University
